# Supplementary material for: Energy requirements and carbon emissions for a low-carbon energy transition
Source: Nat Commun. 2022 Nov 14;13:6932. doi: 10.1038/s41467-022-33976-5 (PMC9663537; doi:10.1038/s41467-022-33976-5)
Supplement: Supplementary file 1 — Supplementary Information [file 41467_2022_33976_MOESM1_ESM.pdf]

SUPPLEMENTARY INFORMATION

Energy requirements and carbon emissions for a low-carbon energy transition

Aljoša Slameršak[1*], Giorgos Kallis[1,2], Daniel W. O'Neill[3]

[1]The Institute of Environmental Science and Technology, ICTA-UAB, Autonomous University of Barcelona, Barcelona, Spain.

[2]Catalan Institution for Research and Advanced Studies, ICREA, Barcelona, Spain.

[3]Sustainability Research Institute, School of Earth and Environment, University of Leeds, Leeds, UK.

[*]e-mail: aljosa.slamersak@gmail.com

Note on EROI$_{FIN}$ estimates from the literature and our study

EROI estimates of different energy generation technologies

Supplementary Figs. 1 and 2 show the EROI$_{FIN}$ values from the literature next to our EROI$_{FIN}$ estimates for the twenty-seven energy conversion technologies used in this study. Besides comparing our EROI calculations to the estimates from the literature, the figures also depict how the EROIs of technologies change over time. Comparing values from the literature to our estimates in the time period from 2020 to 2030 shows that our calculations generally compare closely with the range of literature estimates. The two outliers are the EROIs for wind and photovoltaics (PV), in the period from 2020 to 2030, which are considerably lower in our calculations than in the literature estimates. This difference is because during the initial period of low-carbon energy transition, renewables see an exponential growth in deployment across the range of energy pathways, which results in substantial upfront energy requirements, while most of the energy payback takes place after 2030. The uneven balance between the upfront energy requirements and energy generation translates into a lower EROI of these technologies over the period of rapid growth in their deployment. In the period from 2040 to 2050, when the bulk of renewable infrastructure is already constructed, the EROI of renewables increases substantially due to (a) lower upfront energy requirements as the growth of wind and photovoltaics decrease relative to the energy they generate, (b) low energy requirements for the operation and maintenance of the energy infrastructure, and (c) technological improvements which decrease the energy intensity of construction, operation and maintenance.

Our calculations show that the EROIs of non-CCS fossil fuel and bioenergy technologies at the final energy boundary will decrease in the future both due to higher energy costs of extraction and lower utilisation of these technologies. Depletion of fossil fuels in the most accessible resource extraction sites will lead to a decline in the EROI of fossil fuels at the standard energy system boundary. At the final energy boundary, the EROI of non-CCS technologies decreases below the EROI of CCS technologies in our analysis because conventional fossil fuels, according to the SSP scenario data, become less utilised for energy generation later in the century (supposedly because they are less economic with a rising carbon price). With lower utilisation of fossil fuel technologies, the capacity factors of existing infrastructure decrease, while the phasing-out also comes with an energy cost for the decommissioning of the fossil infrastructure. Both factors result in lower energy return and lower EROI for conventional fossil fuel technologies. This result is the consequence of stranded fossil fuel investments in the future. Some power plants will work at reduced capacities, others will be closed down before the end of their life-time. Thus the “real-world” EROI of these technologies will decline beyond the “real-world” EROI of CCS technologies, which will still operate further into the future, as their carbon intensity is lower in comparison.

As shown by a range of estimates depicted in Supplementary Figs. 1 and 2, alongside Supplementary Table 1, studies may produce different estimates of EROI$_{FIN}$ for a given energy conversion technology. There are three key reasons for different EROI$_{FIN}$ estimates in the literature.

The first is the use of different methodologies to account for energy requirements. EROI methods can omit important energy inputs in the calculation of energy requirements, leading to an overestimation of the $EROI_{FIN}$ of energy conversion technologies[1]. Typically, the process-based methods aim to include all the relevant direct energy inputs to the energy system, such as energy required for construction, decommissioning, and operation and maintenance of energy infrastructure. However, they tend to omit (some) indirect energy inputs, such as the energy required to produce the machinery that is involved in the construction of energy infrastructure or the energy required for exploration of energy resources or to provide for the human labour[2]. In contrast, these indirect energy inputs are captured by input–output methodologies, which are therefore more complete at quantifying total energy requirements.

Comparisons between the two methodological approaches have shown that energy requirements estimated in studies using input–output analysis can exceed the energy requirements estimated in process-based studies by more than 100%[3,4]. However, input–output methodologies require highly detailed data on energy flows between different industries and across countries, which may not always be available and may therefore not be applicable in all case studies. For example, the input–output database used in the EROI study by Brockway et al.[5] describes energy flows between 163 industries across 49 different countries/regions. Such detailed analysis is not possible in our study, which is based on the mitigation pathway energy data from a single energy-system sector of the global economy. For these reasons our method of estimating energy requirements is largely derived from the studies using process-based methodologies like the study by Rauegi and Leccisi[6], which calculate the EROI of power-generation technologies in the United Kingdom.

Second, EROI may vary depending on the specific technological configuration of the energy conversion technology, or depending on the raw resource used, as indicated in Supplementary Table 1. For example, single-crystalline solar panels reportedly have a lower EROI than panels using cadmium telluride[7]. Here, we simplify the calculations of the EROI of photovoltaics (PV) and wind power, which consist of multiple technological configurations, by using a single "representative technology" that aims to capture the average properties of the entire technological space. The energy requirements of the representative PV/wind-power technology are estimated by an inter-quartile range of EROI values from the case studies that feature different technologies listed in Supplementary Table 1.

Third, studies estimate the EROIs of energy conversion technologies in different geographical and temporal settings. Some studies estimate the EROI values of a particular energy generation project, whereas other studies estimate the average regional or global EROI values of a particular energy technology. A study design that quantifies the EROI of the best available technology under the most favourable conditions for energy generation will arrive at higher EROI estimates than a study aiming to quantify the global average EROI of that same technology. For example, the EROI of PV in a mid-latitude country is lower than in a low-latitude country because there is less solar irradiation in the mid-latitudes[8,9].

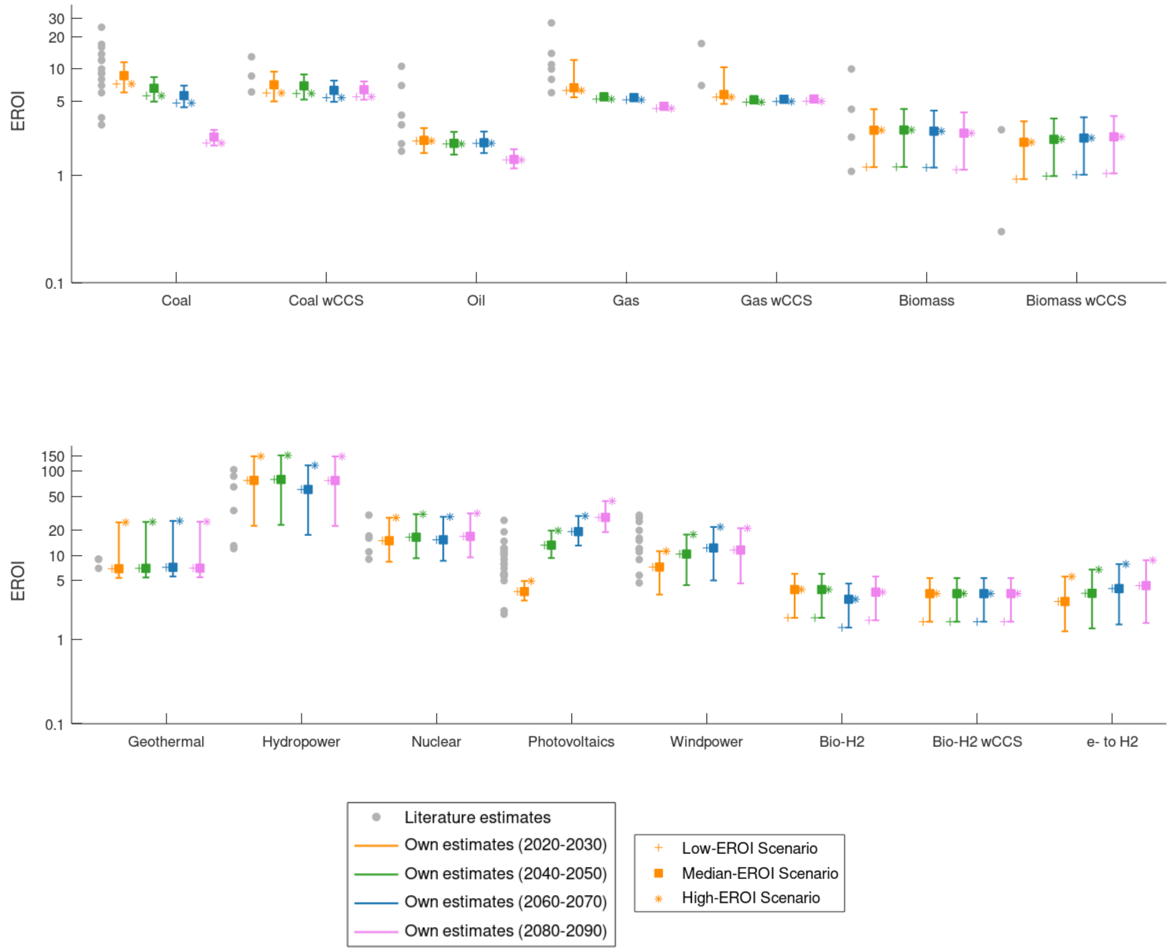

Supplementary Fig. 1: Dynamic EROI*FIN* of power generation and hydrogen technologies. The figure shows our EROI calculations for different power generation technologies and how they change over time. A range of EROI values from the peer-reviewed literature is shown next to our estimates. The full range of estimates is depicted by the error bar. The EROI values of different technologies that are used in our EROI-scenarios are shown with different markers. The depicted EROI values are averaged across fourteen scenarios and over the respective decade as indicated in the legend. For some technologies we did not find any EROI studies in the literature. Abbreviations: wCCS = with carbon capture and storage, Bio-H_2 = hydrogen from biomass, Bio-H_2, wCCS = hydrogen from biomass with carbon capture and storage, e- to H_2 = hydrogen from electrolysis.

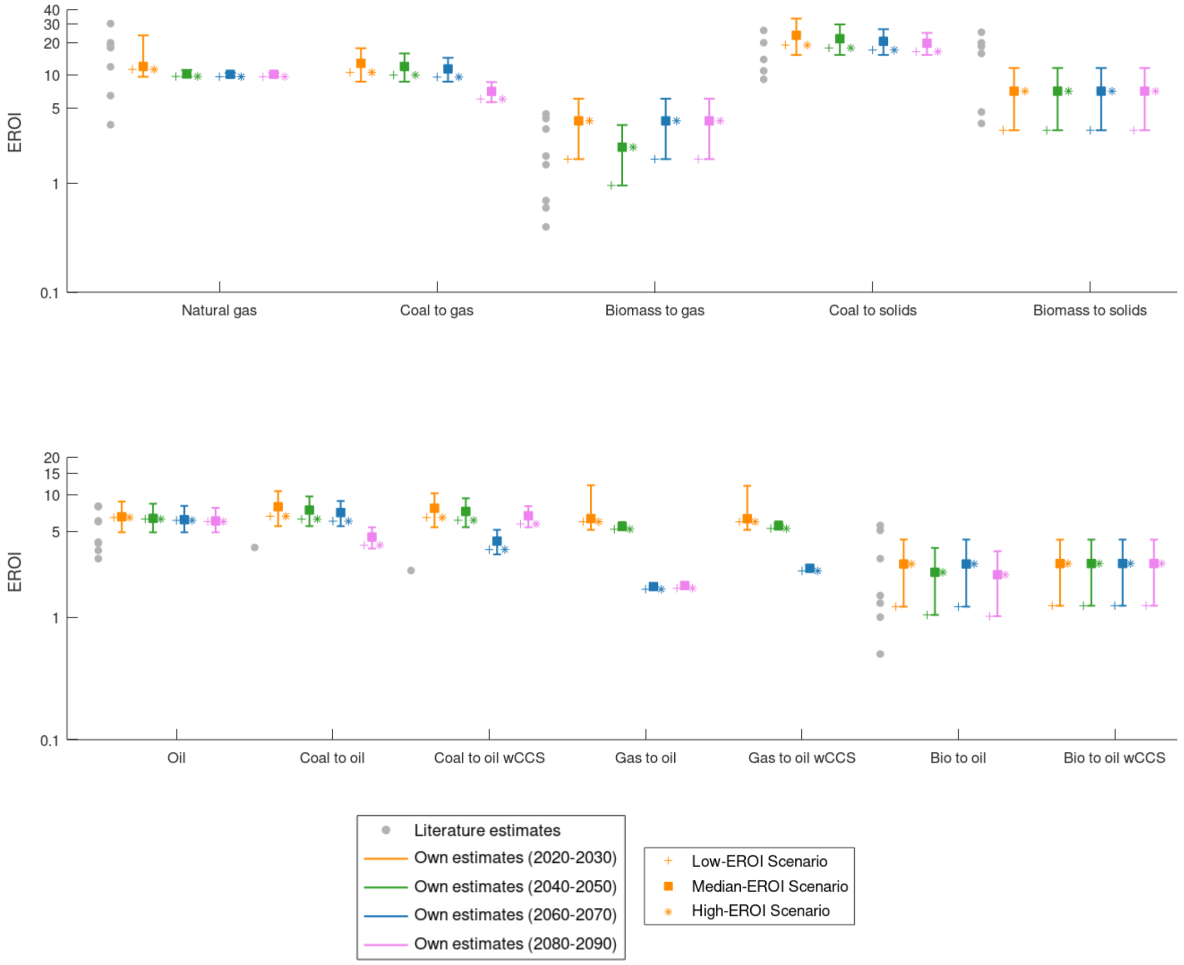

Supplementary Fig. 2: Dynamic EROI$_{FIN}$ of energy conversion into liquids, gases, and solids. The figure shows the range of our EROI calculations for different energy generation technologies and how they change over time. The full range of estimates is depicted by the error bar. The EROI values of different technologies that are used in our EROI-scenarios are shown with different markers. A range of EROI values from the peer-reviewed literature is shown next to our estimates. The depicted EROI values are averaged across fourteen scenarios and over the respective decade as indicated in the legend. For some technologies we did not find any EROI studies in the literature. Abbreviations: wCCS = with carbon capture and storage, Bio to oil = biomass.

Supplementary Table 1: EROI$_{FIN}$ values from literature at the final energy (point-of-use) boundary.

Energy Conversion Technology	EROI$_{FIN}$ Value	Location/Type	Reference
**Coal to electricity**	3	Global[a]	Brockway et al., 2019[5]
	3.5	UK[b]	Raugei and Leccisi, 2016[6]
	7	Chile[b]	Raugei, 2019[1]
	10	Indonesia[b]	Raugei, 2019[1]
	12	Columbia[b]	Raugei, 2019[1]
	6	USA[b]	Raugei and Leccisi, 2016[6]
	8	Global[b]	Kubiszewski, 2010[10]
	9-16	Global[b]	Hall et al., 2014[11]
	9.2-13.8	Global[b]	Sgouridis et al., 2019[12]
	17	Global[b]	King and Van Den Bergh, 2018[13]
	12.2-24.6	Global[b]	Raugei et al., 2012[14]
	6.0-11.9	*Global*	*Our present-day estimate*
**Coal to electricity with CCS**	6.1-8.6	Global[b]	Sgouridis et al., 2019[12]
	13	Global[b]	King and Van Den Bergh, 2018[13]
	4.9-9.5	*Global*	*Our present-day estimate*
**Oil to electricity**	1.7	UK[b]	Raugei and Leccisi, 2016[6]
	2	Chile[b]	Raugei, 2019[1]
	3	Colombia[b]	Raugei, 2019[1]
	7	Global[b]	King and Van Den Bergh, 2018[13]
	3.7-10.6	Global[b]	Raugei et al., 2012[14]
	1.6-2.8	*Global*	*Our present-day estimate*
**Gas to electricity**	11-14	UK[b]	Raugei and Leccisi, 2016[6]
	10	USA[b]	Murphy and Hall, 2010[15]
	27	Global[b]	Sgouridis et al., 2019[12]
	6	Global[b]	Hall et al., 2014[11]
	8	Global[b]	King and Van Den Bergh, 2018[13]
	5.4-15.9	*Global*	*Our present-day estimate*
**Gas to electricity with CCS**	17.3	Global[b]	Sgouridis et al., 2019[12]

	7	Global[b]	King and Van Den Bergh, 2018[13]
	4.7-12.2	*Global*	*Our present-day estimate*
**Biomass to electricity**	1.1	UK[b]	Raugei and Leccisi, 2016[6]
	2.3-4.2	UK[b]	Fajardy and Mac Dowel, 2018[16]
	10	Global[b]	King and Van Den Bergh, 2018[13]
	1.2-4.1	*Global*	*Our present-day estimate*
**Biomass to electricity with CCS**	0.3-2.7[d]	UK[b]	Fajardy and Mac Dowel, 2018[16]
	0.9-3.2	*Global*	*Our present-day estimate*
**Geothermal electricity**	9	Iceland[b]	Atlason and Unnthorsson, 2013[17]
	7	Global[b]	Kubiszewski, 2010[10]
	9	Global[b]	Hall et al., 2010
	7-9	*Global*	*Our present-day estimate*
**Hydropower**	34-87	UK[b]	Raugei and Leccisi, 2016[6]
	12	Global[b]	Kubiszewski, 2010[10]
	65-104	Global[b]	Hall et al., 2014[11]
	13	Global[a]	Castro and Capellan-Perez. 2020[2]
	13-87	*Global*	*Our present-day estimate*
**Nuclear electricity**	30	UK[b]	Raugei and Leccisi, 2016[6]
	9-16	Global[b]	Kubiszewski, 2010[10]
	11-17	Global[b]	Hall et al., 2014[11]
	9-30	*Global*	*Our present-day estimate*
**Photovoltaics[c]**			
cSi	2.2-5.7	UK[b]	Raugei and Leccisi, 2016[6]
cdTe	5.8-14.7	UK[b]	Raugei and Leccisi, 2016[6]
cSi	6-8	Columbia[b]	Raugei and Leccisi, 2016[6]
cdTe	12-19	Columbia[b]	Raugei and Leccisi, 2016[6]
cSi	5-11	USA[b]	Raugei and Leccisi, 2016[6]
cdTe	11-26	USA[b]	Raugei and Leccisi, 2016[6]
	9-10	Switzerland[b]	Raugei et al., 2017[9]
	6	Global[b]	Kubiszewski, 2010[10]

	6-12	Southern Europe[b]	Raugei et al., 2012[14]
	10.4-12.2	Germany[b]	Steffen and Hirscher, 2018[18]
	2.0-5.7	Global[a]	Castro and Capellan-Perez, 2020[2]
	7	Belgium[b]	Limpens and Jeanmart, 2018[19]
	7.8[c]	Global[b]	Louwen et al., 2019[20]
	6.0-9.5	*Global*	*Our present-day estimate*
**Wind power[d]**	15-28	Global (onshore)[b]	Raugei and Leccisi, 2016[6]
	16-30	Global (offshore)[b]	Raugei and Leccisi, 2016[6]
	19.8	Global (operational[b])	Kubiszewski, 2010[10]
	25.2	Germany[b]	Steffen and Hirscher, 2018[18]
	4.7	Global (offshore)[a]	Castro and Capellan-Perez, 2020[2]
	5.8	Global (onshore)[a]	Castro and Capellan-Perez, 2020[2]
	8.9	Global (onshore)[b]	Dupont et al., 2018[21]
	12	Global (offshore)[b]	Dupont et al., 2018[21]
	11-12	Belgium[b]	Limpens and Jeanmart, 2018[19]
	5.8-18.0	*Global*	*Our present-day estimate*
**Biomass to H_2**	*1.8-6.0*	*Global*	*Our present-day estimate*
**Biomass to H_2 with CCS**	*1.6-5.4*	*Global*	*Our present-day estimate*
**Electricity to H_2**	1.7	Global (using PV electricity)[b]	Sathre et al., 2014[22]
	2.3	Global (using PV electricity)[b]	Sathre et al., 2016[23]
	<1.0	Global (using PV electricity)[b]	Hacatoglu et al., 2012[24]
	1.4-6.0	*Global*	*Our present-day estimate*
**Natural gas**	30	UK[b]	Raugei and Leccisi, 2016[6]
	12	USA[b]	Yaritani and Matsushima, 2014[25]
	18	Global[b]	Gagnon et al., 2009[26]
	19	Global[b]	King and Van Den Bergh, 2018[13]
	20	Global[b]	Hall et al., 2014[11]
	3.5-6.5	China[b]	Feng et al., 2018[27]
	9.7-32.2	*Global*	*Our present-day estimate*
**Coal to gas**	*8.7-18.3*	*Global*	*Our present-day estimate*

**Biomass to gas[e]**			
Grass	0.6-3.2	Finland[b]	Usitalo et al., 2017[28]
Barley, Oat and Wheat Ethanol	0.4-1.5	Finland[b]	Usitalo et al., 2017[28]
Wood	0.7-4.0	Finland[b]	Usitalo et al., 2017[28]
Wood	1.8-4.4	Switzerland[b]	Felder and Dones, 2007[29]
	1.7-6.1	*Global*	*Our present-day estimate*
**Oil**	6.1	UK[b]	Raugei and Leccisi, 2016[6]
	3-4	California[b]	Brandt, 2011[30]
	8	Global[a]	Brockway et al., 2019[5]
	4.1	Global[b]	Hall et al., 2009[31]
	8	Colombia[b]	Raugei, 2019[1]
	6	Chile[b]	Raugei, 2019[1]
	3.5-8	China[b]	Feng et al., 2018[27]
	4.9-8.9	*Global*	*Our present-day estimate*
**Coal to oil**	3.7	China[b]	Kong et al., 2015[32]
	5.4-10.5	*Global*	*Our present-day estimate*
**Coal to oil with CCS**	2.4	China[b]	Kong et al., 2015[32]
	5.5-11.0	*Global*	*Our present-day estimate*
**Natural gas to oil**	*5.1-15.9*	*Global*	*Our present-day estimate*
**Natural gas to oil with CCS**	*5.1-10.5*	*Global*	*Our present-day estimate*
**Biomass to oil[e]**			
Ethanol from corn	0.5	USA[b]	Hall et al., 2009[31]
Ethanol from corn	1.0	USA[b]	Murphy et al., 2011[33]
Ethanol from corn	1.3	USA[a,b]	De Castro et al., 2014[34]
Biodiesel	1.5	USA[a,b]	De Castro et al., 2014[34]
Palm oil	3.0	Global[a,b]	De Castro et al., 2014[34]
Ethanol from lignocellulosic feedstock	5.1-5.6	India[b]	Mandade and Shastri, 2019[35]
	1.2-4.3	*Global*	*Our present-day estimate*
**Biomass to oil with CCS**	*1.0-3.7*	*Global*	*Our present-day estimate*

**Biomass to solids**[d]			
Biomass pellets	4.6	UK[b]	Raugei and Leccisi, 2016[6]
Biomass pellets	3.6	UK and EU[b]	Fajardy and Mac Dowel, 2018[16]
Biomass chips	15.9	UK and EU[b]	Fajardy and Mac Dowel, 2018[16]
Biomass chips	18.5-25	Croatia[b]	Pandur et al., 2015[36]
Solid wood	20	Global[b]	Dale et al., 2012[37,38]
	3.1-11.7	*Global*	*Our present-day estimate*
**Coal to solids**	11	UK[b]	Raugei and Leccisi, 2016[6]
	20	Chile[b]	Raugei et al., 2018[39]
	26	Indonesia[b]	Aguirre-Villegas and Benson, 2017[40]
	9.2-14	China[b]	Feng et al., 2018[27]
	15.4-34.7	*Global*	*Our present-day estimate*

Notes: As indicated by location identifier, some studies estimate the $EROI_{FIN}$ of energy infrastructure at a specific location, whereas others aim to quantify the average regional or global $EROI_{FIN}$ of the energy conversion technology.

[a] Studies that use an input–output methodology to quantify energy requirements.

[b] Studies that use a process-based methodology to quantify energy requirements.

[c] Here, we estimate the EROI values of PV by converting the values of "energy payback time", as calculated by Louwen et al.,[20] into the $EROI_{FIN}$. To estimate $EROI_{FIN}$, we divide the expected lifetime of PV by its energy payback time, and multiply it with the conversion factor from primary energy to electricity of 0.311. This approach is consistent with the EROI convention of reporting the sum of energy inputs at the final energy boundary without converting them into primary energy equivalents.

[d] The EROIs of these energy generation technologies can differ depending on the specific technology (PV and wind power) or depending on the raw fuel (biomass type).

[e] Here, we estimate the EROI values of bioelectricity with CCS by converting the values of "electricity return on investment" (E_lROI), as estimated by Fajardy and Mac Dowell[16], into the $EROI_{FIN}$. The authors define the E_lROI as the "ratio of generated electricity to the electrical energy equivalent of energy inputs" (PEeq). To estimate the $EROI_{FIN}$ we divide the PEeq by the average power generation efficiency of the grid, which gives us a first order estimate of total energy requirements. This approach is consistent with the standard EROI convention of summing up all the energy inputs from different energy carriers at the final energy boundary without converting them into primary energy equivalents.

EROI estimates of different energy carriers and the overall energy system

Supplementary Figs. 1 and 2 and Supplementary Table 1 show our calculations of EROI values for different energy generation technologies. In the EROI literature, energy generation technologies are typically grouped together into different energy carriers like liquid fuels and electricity, and energy technology groups (e.g. renewables and fossil fuels), making it possible to analyse the effects of a particular energy policy on the EROI of different parts of the energy system (see Supplementary Table 2).

Supplementary Figs. 3 and 4, and Supplementary Table 2, show our EROI calculations for electricity, liquid fuels, and the overall energy system for the four illustrative pathways, next to the calculations from the literature. The figures show that our model is broadly consistent with the literature in the calculation of present-day EROI values. Only the EROI estimates of fossil fuel carriers from Brockway et al. are different from our present-day EROI estimates. As already explained in the section "EROI estimates of different energy generation technologies", this difference is due to methodological differences between our process-based approach and the input–output approach used by Brockway et al.

The general trend observed in all the pathways is that the EROI of the overall energy system changes little from the present-day values. However, significant changes occur in the EROI of the two main energy carriers. The EROI of liquid fuels declines in the LED, S2-M, and S5-R pathways, as the EROI of oil declines as well as it becomes partly substituted by biofuels, which have comparatively lower EROI. The exception here is S1-A pathway where the EROI of liquid fuels remains unchanged, as this pathway does not consider the substitution of oil for biofuels (Supplementary Fig. 3). Low EROI of liquid fuels after the transition to biofuels means the production of liquid fuels may require similar amounts of energy to the net energy value of fuels produced. The EROI of electricity declines during the initial push for the transition, from 2020 to 2040, when the scale of low-carbon energy infrastructure is expanded dramatically, but increases substantially after 2040. The divergence in the EROIs between liquid fuels and electricity in the long term could incentivise a deeper shift in the economy from liquid fuels to electricity and hydrogen.

Supplementary Fig. 4 shows major differences in the EROI values for electricity between different technology groups. The EROI values of electricity from fossil fuels and low-carbon energy sources, which are comparable in 2020, begin to diverge during the transition. The EROIs of low-carbon electricity increases substantially after 2040, whereas the EROI of fossil fuel electricity gradually declines, due to a declining EROI of fossil fuels at the primary energy stage and a decrease in the use of existing fossil fuels infrastructure. The EROI electricity from bioenergy remains low and may over certain periods drop below the net-energy cliff where EROI = 1.

In future projections, the EROI values vary substantially between different illustrative pathways, which demonstrates the important effect of different endogenous modelling assumptions from different IAMs.

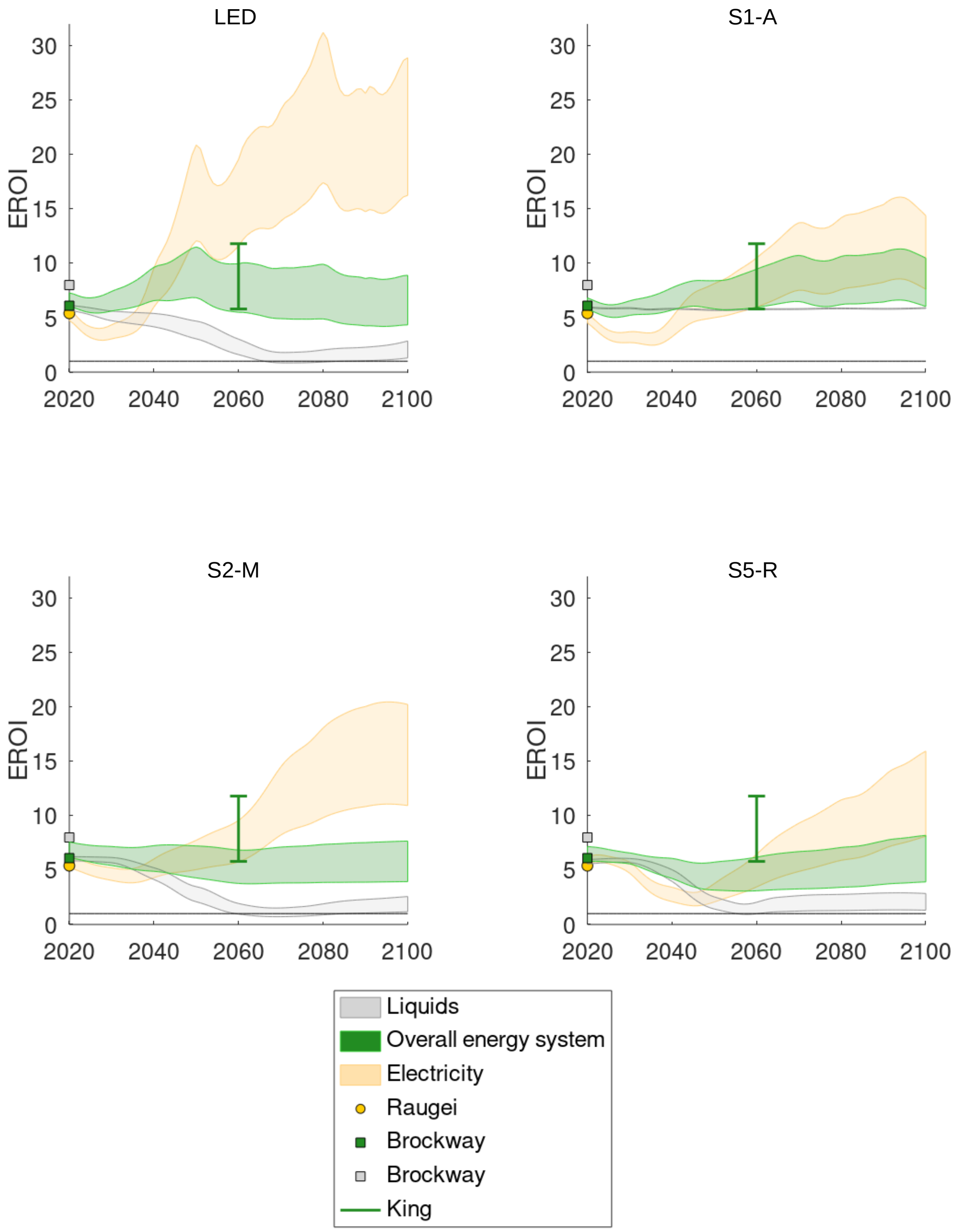

Supplementary Fig. 3: EROI envelopes of different energy carriers and the overall energy system. The figure shows the full range of our EROI estimates for the two main energy carriers, which are electricity and liquid fuels, and the EROI of the overall energy system. The EROI envelopes depict the full scenario space of our EROI trajectories, ranging from the low-EROI to the high-EROI scenario. EROI estimates from the peer-reviewed literature, which are summarised in Supplementary Table 2, are shown next to our estimates. The figure also shows the line where EROI = 1, below which the energy carrier becomes a net energy sink, meaning the amount of energy delivered is lower than the amount of energy required to generate the energy carrier.

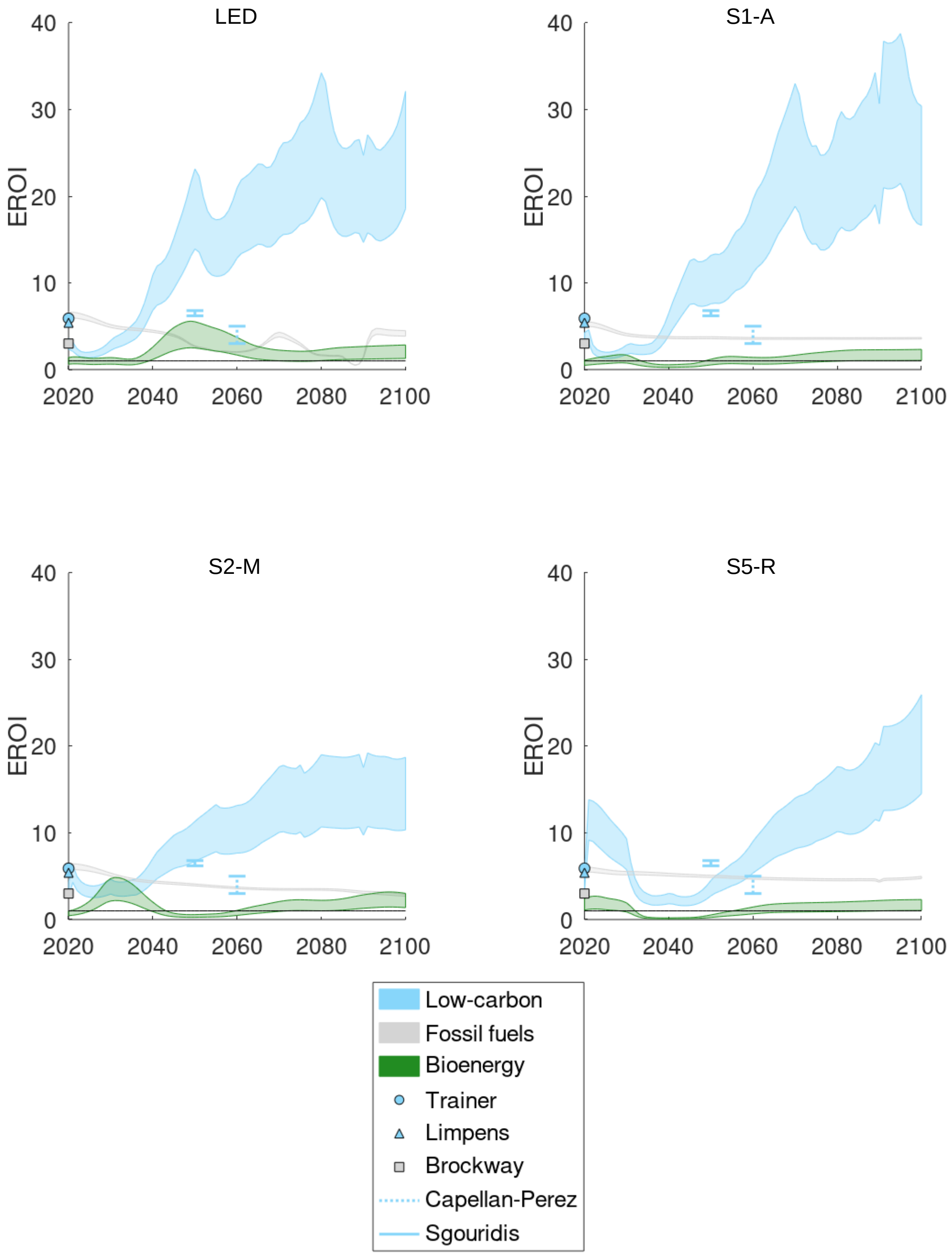

Supplementary Fig. 4: EROI envelopes of electricity from different technology groups. The figure shows the full range of our EROI estimates for different power technologies. The EROI envelopes depict the full scenario space of our EROI trajectories, ranging from the low-EROI to the high-EROI scenario. EROI estimates from the peer-reviewed literature, which are summarised in Supplementary Table 2, are shown next to our estimates. The figure shows the line where EROI = 1, below which the electricity from the technology group becomes a net energy sink, meaning the electricity delivered has a lower energy value than the amount of energy required to generate electricity by the respective technology.

Supplementary Table 2: $EROI_{FIN}$ values from literature at the final energy (point-of-use) boundary.

Energy systems	Scenario assumptions	Estimated $EROI_{FIN}$	Reference
Solar and Wind (electricity only)	100% renewable power system in Australia, backed by storage. The study uses the EROI values of currently available technologies.	5.9	Trainer, 2018[41]
Solar and Wind (electricity only)	100% renewable power globally by 2060 backed up by storage. The study models the evolution of EROI from renewable technologies over time.	From 3 to 5	Capellan Perez et al., 2019[42]
Solar, wind and nuclear (electricity only)	100% low-carbon power system in Belgium, backed up by storage. The study uses the EROI values of currently available technologies.	5.4	Limpens and Jaenmart, 2018[19]
Solar and wind (electricity only)	The study models the EROI in the energy system where 75% of the energy is provided from renewables by 2050.	6.2-6.8*	Sgouridis et al., 2016[43]
Renewables, bioenergy, and nuclear represent approximately 60% of total energy generation, while fossil fuels represent 40%.	Energy system in 2050 from the IEA scenario that is consistent with 66% of staying below 2 °C. The study uses the EROI values of currently available technologies.	From 5.8 to 11.8	King and Van Den Bergh, 2018[13]
Fossil fuels	The study calculates the existing EROI values of energy carriers generated from fossil fuels.	3 (electricity)<br>8 (petroleum and gas)<br>6.1 (all of the energy carriers combined)	Brockway et al., 2019[5]
Fossil fuels represent 66% of power generation, nuclear 19%, biomass 5% and renewables 10%.	UK power grid in 2013.	5.4	Raugei and Leccisi. 2016[6]

* Here, we convert the EROI values from Sgouridis et al.,[43] which are reported in the primary energy equivalent of electrical energy, by dividing the original EROI values ranging from 20-22, by the average power generation efficiency of the UK grid. We use the efficiency of the UK grid from Raugei et al.,[14] which is the original reference for the EROI of renewable energy technologies in the study of Sgouridis et al. This approach is consistent with the standard EROI convention of summing up all the energy inputs from different energy carriers at the final energy boundary without converting them into primary energy equivalents.

Overview of EROI assumptions for different energy conversion technologies

Here, we provide a transparent overview of the qualitative assumptions for the EROI scenarios of different energy technologies across the energy supply chain. Qualitative assumptions provide a rough sketch of the underlying social, political and technological contexts of the development and application of energy technologies in our EROI scenarios.

Supplementary Table 3: **Fossil fuels**

EROI scenario	Extraction	Transportation and distribution	Construction, and operation of infrastructure	Refining of crude oil	Distribution and transmission losses
**High-EROI**	$EROI_{ST}$ median and declining	Long trade routes	Energy required for the construction, and operation is assumed to be equal in all EROI scenarios, as these energy inputs assume only a small share of total energy requirements	Moderate energy intensity	Endogenously accounted in the IAM mitigation pathways
**Median-EROI**	$EROI_{ST}$ median and declining	Moderately long trade routes		Moderate energy intensity	
**Low-EROI**	$EROI_{ST}$ median and declining	Long trade routes		Moderate energy intensity	

Supplementary Table 4: Bioenergy

EROI scenario	Extraction	Transportation and distribution	Construction and operation of infrastructure	Biomass quality	Distribution and transmission losses
**High-EROI**	$EROI_{ST}$ median	Moderately long trade routes	Energy required for the construction, and operation is assumed to be equal in all EROI scenarios, as these energy inputs assume only a small share of total energy requirements	Moderate	Endogenously accounted in the IAM mitigation pathways
**Median-EROI**	$EROI_{ST}$ median	Moderately long trade routes		Moderate	
**Low-EROI**	$EROI_{ST}$ low	Long trade routes		Low	

Supplementary Table 5: Photovoltaics and wind energy

EROI scenario	Construction, operation and maintenance of infrastructure	Resource quality distribution	Distribution, transmission, and storage losses
**High-EROI**	High innovation results in a fast decrease of the energy required to construct, maintain, and operate renewable energy systems	Resource management prioritises high energy yields over other objectives (e.g. conservation of nature reserves), and low public resistance to renewable energy projects result in high energy yields at the sites of most abundant resource density	Endogenously accounted in the IAM mitigation pathways
**Median-EROI**	Moderate innovation results in a moderate reduction of the energy required to construct, maintain, and operate renewable energy systems	Resource management strikes a balance between high energy yields and oteher objectives. Public resistance to renewable energy projects is limited to the areas of high proximity to urban centres and areas of recognised ecological value. These conditions mean that energy yields of energy infrastructure are relatively high, but bellow the maximum tehnical potential	
**Low-EROI**	Moderate innovation results in a moderate reduction of the energy required to construct, maintain, and operate renewable energy systems	Resource management strikes a balance between high energy yields and oteher objectives. Public resistance to renewable energy projects is limited to the areas of high proximity to urban centres and areas of recognised ecological value. These conditions mean that energy yields of energy infrastructure are relatively high, but bellow the maximum tehnical potential	

Supplementary Table 6: **Nuclear energy, hydropower, and geothermal energy**

EROI scenario	EROI assumptions	Distribution and transmission losses
**High-EROI**	The upper quartile of present-day EROI values	Endogenously accounted in the IAM mitigation pathways
**Median-EROI**	Median of present-day EROI values	
**Low-EROI**	The lower quartile of present-day EROI values	

Supplementary Table 7: Hydrogen from electrolysis

EROI scenario	Energy system conversion efficiency	Distribution and transmission losses
**High-EROI**	High innovation leads to very high efficiency improvements of electrolysis and reduced energy requirements of a hydrogen fuel cell	Endogenously accounted in the IAM mitigation pathways
**Median-EROI**	Moderate innovation results in high efficiency improvements of electrolysis and lower energy requirements of a hydrogen fuel cell	
**Low-EROI**	High innovation leads to very high efficiency improvements of electrolysis and reduced energy requirements of a hydrogen fuel cell	

Supplementary Table 8: The fourteen scenarios used in our study

Scenario Narrative	Integrated Assessment Model	Abbreviations
LED - Low Energy Demand[44,45]	MESSAGE GLOBIOM	LED
S1- Sustainable Development[46–48]	IMAGE	S1-I
	AIM	S1-A
	GCAM4	S1-G
	MESSAGE GLOBIOM	S1-M
	REMIND MAgPIE	S1-R
	WITCH	S1-W
S2 – Middle of the Road[46,47,49]	AIM	S2-A
	GCAM4	S2-G
	MESSAGE GLOBIOM	S2-M
	REMIND MAgPIE	S2-R
S4 – World of deepening Inequality[46,47,50]	WITCH	S4-W
S5 – Fossil-fuelled development[46,47,51]	GCAM4	S5-G
	REMIND MAgPIE	S5-R

Note: The table provides a list of fourteen scenarios used in our study and their abbreviations. We group the scenarios according to their scenario narratives. Scenario narratives underpin the Shared Socioeconomic Pathways (SSPs) and outline different socioeconomic developments. SSP narratives lay out basic assumptions on technology developments, lifestyle changes, and resource availability. The assumptions from SSPs are then used in integrated assessment models (IAMs) to produce the mitigation pathways that are compatible with the climate target of stabilising climate change below 1.5 °C by 2100. LED is a scenario that was produced after the release of the SSPs and is based on a "low-energy narrative" that is distinct from any of the SSP narratives.

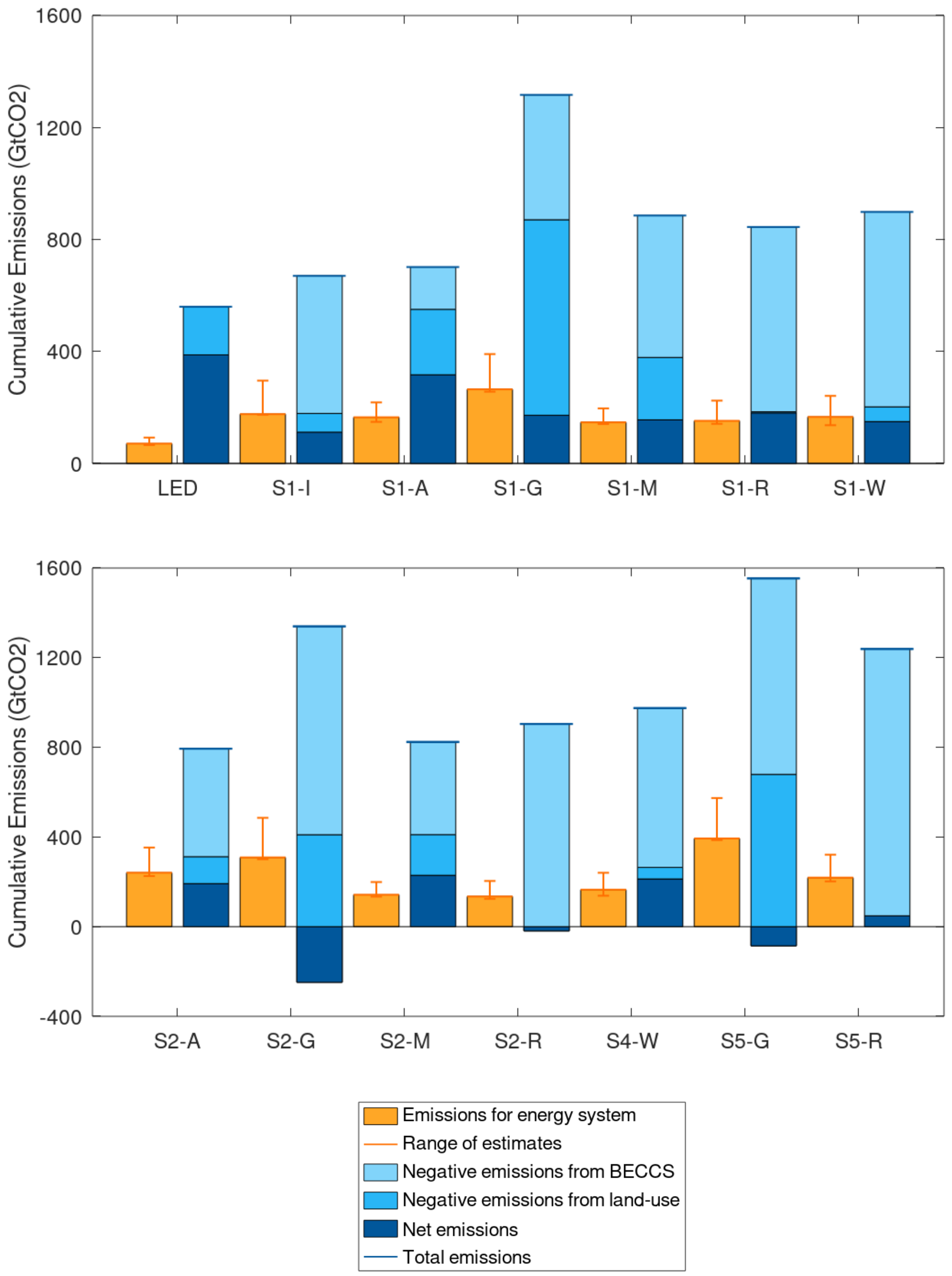

Supplementary Fig. 5: Energy system emissions for the fourteen scenarios compatible with 1.5 °C. Energy system emissions (orange columns) are compared to total cumulative emissions (blue columns). Orange error bars indicate the spread of energy system emissions calculations from high- to low-EROI scenarios.

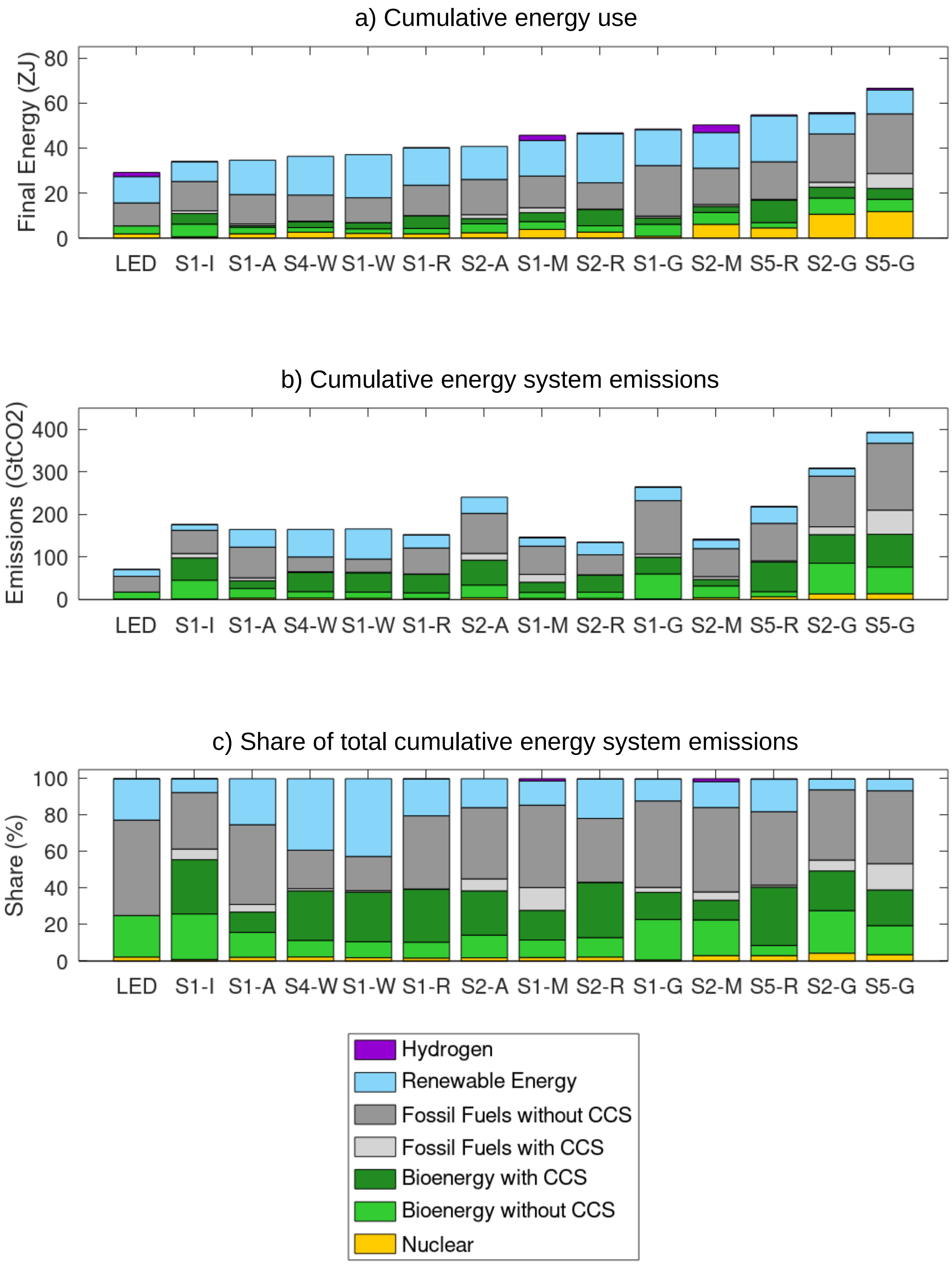

Supplementary Fig. 6: Cumulative energy use and energy system emissions from different energy technologies for scenarios compatible with 1.5 °C. (a) Total amount of energy consumption in the period from 2020 to 2100. Individual technologies are aggregated together depending on the energy generation type. Scenarios in all the panels are ordered by energy consumption. **(b)** Total amount of energy system emissions for the median EROI assumption. **(c)** The share of emissions for the energy system from each energy source for the median EROI assumption.

Notes: The speed of decarbonisation in different scenarios can be approximated from the cumulative energy system emissions from fossil fuels without carbon capture and storage (Panel a). The speed of decarbonisation is roughly speaking inversely proportional to the cumulative use of fossil fuels without CCS (Panel b). Note that BECCS produces a relatively small amount of energy (Panel a), but represents a major source of energy system emissions in the majority of scenarios (Panels b and c).

Supplementary Table 9: Panel data analysis of the factors that drive energy system emissions

	Annual Energy System Emissions ($GtCO_2$/year)	
	**2020–2040**	**2041–2100**
Energy Use (EJ/year)	0.0081***	0.0042***
	(9.714e-05)	(0.0002)
Share of Conventional Fossil Fuels (%)	0.0120***	0.0896***
	(0.0018)	(0.0026)
1/EROI (%)	0.1397***	-0.0436***
	(0.00413)	(0.0096)
Constant	-2.7540***	-0.0069***
	(0.0544)	(0.0013)
Number of Observations	294	840
R^2 (Between Scenarios)	0.992	0.880

Note: We performed an OLS regression with time fixed-effects on average annual energy system emissions for three energy system emissions factors. We conducted separate analyses for the 2020–2040 and 2041–2100 periods. Robust standard errors are shown in parentheses. *** $p < 0.001$.

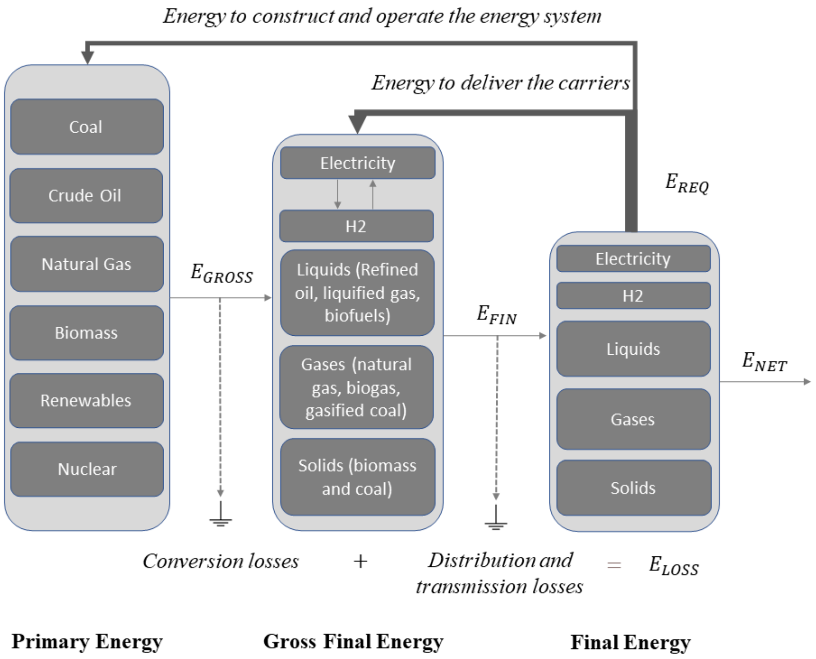

Supplementary Fig. 7: Boundaries of net energy analysis and energy flows along the energy supply chain. The figure illustrates how different technologies convert energy from the primary energy stage to the final energy stage, where net energy is delivered to society. At the primary energy stage, raw resources are extracted, or harvested, before being sent to energy conversion or energy processing facilities like power plants and refineries at the secondary energy stage. This is the energy system boundary for calculating the EROI at the standard energy system boundary ($EROI_{ST}$). The secondary energy stage is where most of the useful energy carriers are generated. The total amount of energy generated at this stage is known as the gross final energy. A fraction of gross final energy is "lost" during the distribution and transmission to end users. A fraction of the remaining final energy is used by the energy system itself (e.g. for extraction, conversion, and the delivery of energy to the end users). Some of the final energy is also used by the industry to produce new energy infrastructure that replaces the obsolete infrastructure. The remaining energy that goes to society is defined as net energy.

Supplementary Table 10: Model parameter values

Parameter	Abbreviation	Specification	Value (min-max)	Unit	Reference
Energy intensity of capital	ε		4520	GJ/million $US2015	Sgouridis et al., 2019[12]
Capital costs of infrastructure	C_p			$/ per kW of installed capacity	
		Coal to electricity	2200		REMIND[52,53]
		Coal to electricity wCCS	2800		REMIND[52,53]
		Oil to electricity	1000		Our assumption
		Gas to electricity	950		REMIND[52,53]
		Gas to electricity wCCS	1350		REMIND[52,53]
		Biomass to electricity	2450		REMIND[52,53]
		Biomass to electricity wCCS	3150		REMIND[52,53]
		Natural gas	0[a]		Our assumption
		Coal to gas	1440		REMIND[52,53]
		Biomass to gas	1200		REMIND[52,53]
		Oil	0[a]		Our assumption
		Coal to oil	1740		REMIND[52,53]
		Coal to oil wCCS	1820		REMIND[52,53]
		Gas to oil	1030		Larson et al., 2012[54]
		Gas to oil wCCS	1230		Larson et al., 2012[54]
		Biomass to oil	3000		REMIND[52,53]
		Biomass to oil wCCS	3600		REMIND[52,53]
		Coal to solids	0[a]		REMIND[52,53]
		Biomass to solids	0[a]		REMIND[52,53]
		Biomass to H_2	1680		REMIND[52,53]
		Biomass to H_2 wCCS	2040		REMIND[52,53]
Operation and Maintenance costs	$C_{O\&M}$			$/ per GJ of generated energy	
		Coal to electricity	4.0		REMIND[52,53]
		Coal to electricity wCCS	5.3		REMIND[52,53]
		Oil to electricity	7.0		Our assumption
		Gas to electricity	2.1		REMIND[52,53]
		Gas to electricity wCCS	2.9		REMIND[52,53]
		Biomass to electricity	5.1		REMIND[52,53]
		Biomas to electricity wCCS	6.9		REMIND[52,53]
		Natural gas	0[a]		Our assumption

		Coal to gas	1.4		REMIND[52,53]
		Biomass to gas	1.9		REMIND[52,53]
		Oil	0		Our assumption
		Coal to oil	4.2		REMIND[52,53]
		Coal to oil wCCS	5.0		REMIND[52,53]
		Gas to oil	1.2		Our assumption
		Gas to oil wCCS	1.5		Our assumption
		Biomass to oil	4.2		REMIND[52,53]
		Biomass to oil wCCS	5.4		REMIND[52,53]
		Coal to solids	0[a]		Our assumption
		Biomass to solids	0[a]		Our assumption
		Biomass to H_2	5.7		REMIND[52,53]
		Biomass to H_2 wCCS	6.8		REMIND[52,53]
Rate of decay	β_C			% per year	
		Coal		2.2	Brockway et al., 2019[5]
		Gas		1.6	Brockway et al., 2019[5]
		Oil		1.1	Brockway et al., 2019[5]
Energy conversion efficiency	η_C			Dimensionless	
		Coal to electricity	(0.41-0.50)[b]		REMIND[52,53]
		Coal to electricity wCCS	(0.33-0.43)[b]		REMIND[52,53]
		Oil to electricity	0.35		IPCC, 2007[55]
		Gas to electricity	(0.56-0.63)[b]		REMIND[52,53]
		Gas to electricity wCCS	(0.49-0.56)[b]		REMIND[52,53]
		Biomass to electricity	(0.37-0.46)[b]		REMIND[52,53]
		Biomas to electricity wCCS	(0.28-0.35)[b]		REMIND[52,53]
		Natural gas	1.0[c]		Our assumption
		Coal to gas	0.6		REMIND[52,53]
		Biomass to gas	0.55		REMIND[52,53]
		Oil	1.0[c]		Our assumption
		Coal to oil	0.40		REMIND[52,53]
		Coal to oil wCCS	0.40		REMIND[52,53]
		Gas to oil	(0.53-0.58)[b]		Larson et al., 2012[54]
		Gas to oil wCCS	(0-53-0.58)[b]		Larson et al., 2012[54]
		Biomass to oil	0.40		REMIND[52,53]

		Biomass to oil wCCS	0.41		REMIND[52,53]
		Coal to solids	1.0[c]		Our assumption
		Biomass to solids	1.0[c]		Our assumption
		Biomass to H_2	0.61		REMIND[52,53]
		Biomass to H_2 wCCS	0.55		REMIND[52,53]
Energy intensity of oil refinery per kg output	μ_{REF}		3.8[d] (2.8[d] -4.5)[d]	MJ/kg	Meili et al., 2018[56] Jing et al., 2020[57] Raugei and Leccisi, 2016[6]
Higher heating value	HHV			MJ/kg	
		Coal	25.2		Raugei and Leccisi, 2016[6]
		Natural gas	38.3		Raugei and Leccisi, 2016[6]
		Oil (petroleum)	45.8		Raugei and Leccisi, 2016[6]
		Biomass – miscanthus and wheat straw	18.4 (17.3-21.2)[e]		Fajardy and Mac Dowel, 2017[58]
Energy intensity of transportation per tonne km	$\gamma_{TRA,j}$			MJ/tkm	
		Shipping (bulk)	0.11		EcoInvent v3.2[59]
		Shipping (tanker)	0.06		EcoInvent v3.2[59]
		Shipping (LNG)	0.37		EcoInvent v3.2[59]
		Oil pipeline offshore	0.63		EcoInvent v3.2[59]
		Oil pipeline onshore	0.18		EcoInvent v3.2[59]
		Gas pipeline offshore	0.35		EcoInvent v3.2[59]
		Gas pipeline onshore	0.35		EcoInvent v3.2[59]
		Truck >32tonnes	1.26		EcoInvent v3.2[59]
		Truck >16tonnes	1.93		EcoInvent v3.2[59]
		Rail	0.14		EcoInvent v3.2[59]
		Barge	0.52		EcoInvent v3.2[59]
Lifetime of infrastructure	τ			years	
		Coal to electricity	35		REMIND[52,53]
		Coal to electricity wCCS	35		REMIND[52,53]
		Oil to electricity	35		Our Assumption
		Gas to electricity	35		REMIND[52,53]
		Gas to electricity wCCS	35		REMIND[52,53]
		Biomass to electricity	40		REMIND[52,53]

		Biomas to electricity wCCS	40		REMIND[52,53]
		Natural gas	35		REMIND[52,53]
		Coal to gas	35		REMIND[52,53]
		Biomass to gas	35		REMIND[52,53]
		Oil	30		CAPP[60]
		Coal to oil	35		REMIND[52,53]
		Coal to oil wCCS	35		REMIND[52,53]
		Gas to oil	35		Larson et al., 2012[54]
		Gas to oil wCCS	35		Larson et al., 2012[54]
		Biomass to oil	35		Our assumption
		Biomass to oil wCCS	35		Our assumption
		Coal to solids	45		King and Van Den Bergh, 2018[13]
		Biomass to solids	35		Own assumption
		Biomass to H_2	35		REMIND[52,53]
		Biomass to H_2 wCCS	35		REMIND[52,53]
		Geothermal power	30		REMIND[52,53]
		Hydropower	70		REMIND[52,53]
		Nuclear power	40		REMIND[52,53]
		Photovoltaics	30		REMIND[52,53]
		Windpower	25		REMIND[52,53]
		Electricity to H_2	30		Own assumption
Operation share of energy requirements	α_{tech}			Dimensionless	
		Geothermal power	0.95		Atlason et al., 2013[17]
		Hydropower	0		Arvesen et al., 2018[61]
		Nuclear power	0.904		Arvesen et al., 2018[61]
		Photovoltaics	0.01		Arvesen et al., 2018[61]
		Windpower	0.1215[f]		Arvesen et al., 2018[61]
		Electricity to H_2	1.0		Our assumption
Experience parameter	b			Dimensionless	
		Photovoltaics	0.235 (0.193-0.278)[g]		Steffen et al., 2018[18]
		Windpower	0.015 (-0.036-0.066)[g]		Steffen et al., 2018[18]
Energy stored on energy invested	ESOI	Hydrogen fuel cell	59 (65-68)[h]	Dimensionless	Pellow et al., 2015[62]

Electrolyser efficiency	η_{lyz}		0.78 (0.70-0.85)[h]	Dimensionless	Pellow et al., 2015[62]
Fuel cell efficiency	η_{FC}		0.60 (0.47-0.72)[h]	Dimensionless	Pellow et al., 2015[62]
Hydrogen compression efficiency	η_{comp}		0.89 (0.93-0.96)[h]	Dimensionless	Pellow et al., 2015[62]
Full H_2 system efficiency	η_{sys}		0.442 (0.303-0.591)[h]	Dimensionless	Pellow et al., 2015[62]
Carbon intensity of energy conversion technologies	φ_i			tCO_2/GJ	
		Coal	0.0957		REMIND[52,53]
		Coal wCCS	0.00957		REMIND[52,53]
		Oil	0.0675		REMIND[52,53]
		Gas	0.0561		REMIND[52,53]
		Gas wCCS	0.00561		REMIND[52,53]
		Biomass	0		REMIND[52,53]

Notes: [a] Capital costs and operation and maintenance costs of energy infrastructure for indicated technologies are already accounted for in $EROI_{ST}$ at the standard boundary, therefore the value is 0.

[b] Minimum values of energy conversion efficiencies represent the technological efficiencies of energy infrastructure built in year 2005, whereas maximum values correspond to the efficiencies of infrastructure build after 2050, as assumed in the REMIND IAM documentation. For energy conversion efficiencies of energy infrastructure build between 2005 and 2050, we use a linear interpolation between the values of 2005 and 2050.

[c] Energy conversion efficiencies equal 1.0 when there is no energy conversion loss. We assume zero losses for natural gas and oil, as we use the data at the secondary energy stage which already accounts for conversion losses. We also assume zero losses for biomass and coal, which is consistent with the energy conversion in the majority of mitigation scenarios that were analysed.

[d] Lower value for energy intensity of oil refinery are taken from Meili et al.,[56] median value is taken from Jing et al.,[57] and the high value is taken from Raugei and Leccisi[6].

[e] The range of HHV values for biomass is taken from the HHV values of miscanthus, and wheat straw, according to Fajardy and Mac Dowel[16].

[f] Here, the operation share of energy requirements for wind power is estimated as an average of operation shares for offshore and onshore wind-power, calculated by Arvensen et al[61].

[g] Experience parameters for PV and Wind power are calculated following the approach of Steffen et al[18]. We calculate the experience parameter b from the "invested energy data" provided in Figure 1 of the latter study. In the calculation of experience parameters, we do not account for the improved efficiency of energy generation, as these improvements and their effects on the EROI are already endogenously included in the energy generation data from the mitigation scenarios.

[h] Parameters of hydrogen generation from electrolysis were obtained from the Table 1 (compressor efficiency) and Table 3 (ESOI, electrolyser efficiency, and fuel cell efficiency) of the study by Pellow et al[62]. The system efficiency parameter was calculated using the Supplementary Equation 11 from the "Note on energy requirements of hydrogen from electrolysis", which was derived from the same study.

Note on EROI$_{ST}$ estimates from literature

We constructed a database of EROI values for different energy fuels at the standard energy system boundary by conducting a Google Scholar search using the following queries: "EROI + fuel type" (e.g. "EROI + biomass"), and "net-energy analysis". We collected all studies containing EROI calculations for the raw fuels included in our study. Furthermore, we manually checked the studies cited in the relevant EROI review literature[11,33,38,63,64].

Of the 39 studies obtained, we eliminated studies with ambiguous EROI methodologies or boundaries that did not fit those defined in our study. We also excluded studies that used EROI estimates originating from studies we had already selected, to avoid double-counting. EROI data from the remaining 23 studies, listed in Supplementary Table 10, were used for the interquartile analysis producing low, median, and high-EROI$_{ST}$ estimates for the raw energy fuels in our model. The EROI$_{ST}$ values are used to calculate the energy required for the extraction, mining, or harvesting of raw fuels and should not be confused with the EROI$_{FIN}$ estimates reported in Supplementary Table 1, which include additional energy requirements for the conversion of raw fuels into useful energy carriers and their delivery to consumers at the final energy stage.

Supplementary Table 11: Overview of EROI$_{ST}$ estimates from the literature at the standard energy system boundary alongside our calculations

Energy resource	EROI$_{ST}$ value (min/median/max)	Location/Origin	Reference
Coal			
	46	USA	Raugei and Leccisi, 2016[6]
	60	Colombia	Raugei and Leccisi, 2016[6]
	27	UK	Raugei and Leccisi, 2016[6]
	18	Russia	Raugei and Leccisi, 2016c
	40-55	Global	Hall et al., 2014[11]
	60	USA	Hall et al., 2014[11]
	27	China	Hu et al., 2013[65]
	80	USA	Murphy and Hall, 2010[15]
	42	Global	Dale et al., 2012[37,38]
	28	Global	Lambert et al., 2012[63]
	42	Indonesia	Aguirre-Villegas and Benson, 2017[40]
	65	Chile	Raugei et al., 2018[39]
	23-58	Global	Sgouridis et al., 2019[12]
	29	Global	Brockway et al., 2019[5]
	**27/42/59**	**Global IQ-range[a]**	**Our calculation**
Oil	86	UK	Raugei and Lecissi, 2016[6]
	10	Algeria	Raugei and Lecissi, 2016[6]
	6	Nigeria	Raugei and Lecissi, 2016[6]
	49	Norway	Raugei and Lecissi, 2016[6]
	9	China	Hu et al., 2013[65]
	25	Colombia	Yañez et al., 2018[66]
	24	Chile	Raugei et al., 2018[39]
	18	Global	Cleveland, 2011[67]
	13	Canada	Poisson and Hall, 2013[68]
	11	USA	Guiford et al., 2011[69]
	10-20	USA	Murphy et al., 2011[33]

	24	Global	Dale et al., 2012[37,38]
	17	Global	Lambert et al., 2012[63]
	18	Global	Gagnon et al., 2009[26]
	20	Global	Hall et al., 2014[11]
	28	Global	Brockway et al., 2019[5]
	**11/18/24**	**Global IQ-range[a]**	**Our calculation**
Natural Gas			
	78	UK	Raugei and Lecissi, 2016[6]
	115	Norway	Raugei and Lecissi, 2016[6]
	294	Netherlands	Raugei and Lecissi, 2016[6]
	87	USA	Sell et al., 2011[70]
	17	USA	Yaritani and Matsushima, 2014[25]
	10	USA[b]	Murphy et al., 2011[33]
	20	Canada[b]	Lambert et al., 2012[63]
	9	China[b]	Hu et al., 2013[65]
	13	Canada[b]	Poisson and Hall, 2013[68]
	20	Canada[b]	Lambert et al., 2012[63]
	11	USA[b]	Guilford et al., 2011[69]
	53	Global	Sgouridis et al., 2019[12]
	20	Global[b]	Hall et al., 2014[11]
	18	Global[b]	Gagnon, 2009[26]
	29	Global	Brockway et al., 2019[5]
	**13/20/78**	**Global IQ-range[a]**	**Our calculation**
**Biomass[c]**			
Pellets	3.1	USA	Raugei and Lecissi, 2016[6]
Chips	54	UK	Raugei and Lecissi, 2016[6]
Straw	4.5	UK	Raugei and Lecissi, 2016[6]
Solid biomass	20	Global	Dale et al., 2012[37,38]
Chips	30	Croatia	Pandur et al., 2015[36]
Chips	14.4	North Europe	Moriarty et al., 2016[71]

Wheat pellets	7.8/9.9/13.5[d]	EU	Fajardy and Mac Dowel, 2017[58]
Switchgrass pellets	5.0/9.0/14.8[d]	EU	Fajardy and Mac Dowel, 2017[58]
Miscanthus pellets	3.8/5.9/10.3[d]	EU	Fajardy and Mac Dowel, 2017[58]
Willow pellets	2.1/2.5/2.7[d]	EU	Fajardy and Mac Dowel, 2017[58]
Wheat pellets	7.8/9.5/13.5[d]	USA	Fajardy and Mac Dowel, 2017[58]
Switchgrass pellets	5.0/8.1/14.8[d]	USA	Fajardy and Mac Dowel, 2017[58]
Miscanthus pellets	3.8/5.6/10.3[d]	USA	Fajardy and Mac Dowel, 2017[58]
Willow pellets	1.5/2.1/2.7[d]	USA	Fajardy and Mac Dowel, 2017[58]
Wheat pellets	5.6/9.4/19.8[d]	Brazil	Fajardy and Mac Dowel, 2017[58]
Switchgrass pellets	3.5/7.2/28.7[d]	Brazil	Fajardy and Mac Dowel, 2017[58]
Miscanthus pellets	3.0/5.8/18.6[d]	Brazil	Fajardy and Mac Dowel, 2017[58]
Wheat pellets	4.5/8.5/24.1[d]	China	Fajardy and Mac Dowel, 2017[58]
Miscanthus pellets	3.2/13.3[d]	China	Fajardy and Mac Dowel, 2017[58]
Switchgrass pellets	4.2/9.5/67.7[d]	China	Fajardy and Mac Dowel, 2017[58]
Wheat pellets	4.3/9.5/13.1[d]	India	Fajardy and Mac Dowel, 2017[58]
Miscanthus pellets	2.3/4.3/13.1[d]	India	Fajardy and Mac Dowel, 2017[58]
Switchgrass pellets	2.2/5.4/25[d]	India	Fajardy and Mac Dowel, 2017[58]
Dry Switchgrass	23	Canada	Hall et al., 2011[72]
Dry Switchgrass	38	USA	Hall et al., 2011[72]
Verge grass (natural drying)	45-60	Netherlands	Voinov et al., 2015[73]
Willow chips (natural drying)	83-102	Netherlands	Voinov et al., 2015[73]
**Chips**	**20/26/38**	**Global IQ-range[a]**	**Our calculation**
**Pellets**	**1.9/5.5/9.2**	**Global IQ-range[a]**	**Our calculation**
**50% pellets, 50% chips**	**3.6/9.1/14.9**	**Global IQ-range[a]**	**Our calculation**

Notes: [a] We use the range of EROI values of each energy resource from the literature to calculate the inter-quartile range of EROI values (IQR), consisting of: lower-quartile, median, and higher-quartile EROI values of the resource. We use these estimates in the model to calculate the energy requirements for the extraction, mining or harvesting of energy resources (raw fuels) before they are converted into useful energy carriers.

[b] EROI values for a combined extraction of oil and natural gas. Extraction of natural gas commonly takes place alongside extraction of crude oil, therefore, many studies analyse energy inputs associated with the extraction of oil and natural gas together and report a single EROI value for both fuels.

[c] EROI values of biomass include energy requirements of harvesting at the standard system boundary, and energy inputs associated with processing, drying, and chipping or pelleting of biomass.

[d] EROI estimates from Fajardy and Mac Dowel were adjusted by deducing from energy requirements the energy used in the transportation of biomass. This was done to avoid the double counting of energy used in transportation when calculating the EROI of energy conversion technologies from biomass using our method.

Supplementary Table 12: Energy requirements of the global transportation of liquid fuels, estimated from the analysis of major global trade routes of crude oil and oil products.

Trade Route[a]	Distances (tkm)				Trade flow in million tonnes and (%)[f]	Energy intensity of transport (MJ/kg)	References
	Onshore pipeline[b]	Offshore pipeline[c]	Sea freight[d]	Truck freight[e]			
Canada (Edmonton)– USA (Pine Bend in Minnesota)	1.88	0	0	0.1	269.4 (7.7%)	0.47	NRCAN[74]
Middle East (Bagdad via. Ceyhan) - USA (Houston)	1.07 (0.97 in the Middle East and 0.1 in Houston)	0	12.5	0.1	52.8 (1.5%)	1.07	Meili et al., 2018[56]
West-Africa (Onne) - USA (Houston)	0.24 (0.14 in Nigeria and 0.1 in USA)	0.02	11	0.1	33.8 (1.0%)	0.85	Meili et al., 2018[56]
Mexico (Altamira)-US (Houston)	0.2	0.2	0.9	0.1	32.3 (0.9%)	0.35	Meili et al., 2018[56]
Brazil (Sao Paulo)-China (Quingdao)	0.1	0.2	20.5	0.1	68.5 (2.0%)	1.50	SeaRates[75]
Russia (Taishet)- China (Skovorodino)	3.8	0	0	0.1	80.8 (2.3%)	0.81	Global energy monitor[76]
Middle East (Abqaiq) – China (Shenzen)	0.1	0.02	9.4	0.1	244.8 (7.0%)	0.73	Sea Rates[75]
West-Africa (Onne) - China (Shenzen)	0.14	0.02	17.3	0.1	91.8 (2.6%)	1.21	Sea Rates[75]
Middle East (Mina via. Al-Ahmadi) - New Mangalore	0.1	0	3.6	0.1	158.9 (4.6%)	0.36	Sea Rates[75]
West-Africa (Onne) - India (Jamnagar)	0.14	0.02	13.7	0.1	47.6 (1.4%)	0.99	Sea Rates[75]
Middle East (Dubai) - Japan (Yokohama)	0.1	0	11.8	0.1	141.1 (4.1%)	0.86	Sea Rates[75]
Middle East (Abqaiq) - Singapore	0.1	0.02	8	0.1	56.7 (1.6%)	0.64	Sea Rates[75]
Long-distance oil transport to Europe	2	0.04	2.2	0.1	731.7 (21.0%)	0.65	Meili et al., 2018[56]

Average energy for domestic transport[g]	0.6	0.04	0	0.1		0.26	Our calculation
**Weighted average energy intensity of oil transport**					**3480.9 (100%)**	**0.61+/-0.11**[f]	

Notes: [a] Selected trade routes represent some of the major destinations of global export and import of crude oil. We divide the trade routes into four transportation segments (onshore pipeline, offshore pipeline, freight by tankers, and freight by truck with the cargo capacity of 32 tonnes). Our approach broadly follows the methods and assumptions of Meili et al.[56]

[b] For distances of oil transport in pipelines we used the information on existing pipeline networks that was available in online documentation of pipeline networks, unless the distances were already provided in the study by Meili et al.[56] For trade routes where oil is delivered to the refineries that are situated nearby the ports, we assume the onshore pipeline value of 100 km.

[c] For trade routes where offshore oil fields represent the main share of oil extraction, we assume a generic offshore pipeline value of 200 km, whereas for trade routes where offshore represents a small share of oil extraction, we assume 20 km, following Meili et al.[56]. For oil exports from oilwells based on the mainland, we assume the distance of offshore pipelines to be zero.

[d] For transportation across the open sea, we used the application "Sea Rates[75]" which calculates the distances between ports.

[e] We assume an average global distance of delivery by a truck from the refinery to the final user of 100km.

[f] We match the selected trade routes with the volume of transported crude oil and oil products obtained from "Oil: Inter-area movements 2019" input and output table from the British Petroleum's (BP) *"Statistical Review of World Energy 2020"*[77]. According to the BP data tables on global oil production and trade, the selected trade routes transport 58% of globally produced crude oil and oil products. We calculate relative shares of global oil production that is transported over a selected trade route and use them as relative weights to calculate the average global energy requirement for transporting liquid fuels, as shown in Equation 13 of the Methods. The standard error of our global intensity estimate is assumed to be the double of the standard deviation of the energy intensities in the selected trade routes.

[g] Domestic transport of oil corresponds to the 22.4% of global oil production for which the extraction, refining and end-use take place within the same country. For domestic oil transport, we assume an average onshore pipeline distance of 600 km, which is consistent with the average distance of the domestic oil transport in pipelines in the USA[78], an average offshore pipeline distance of 40 km as suggested for Europe by Meili et al.,[56] and 0 km of sea.

Supplementary Table 13: Energy requirements of the global transportation of natural gas, estimated from the analysis of major global trade routes of natural gas.

Trade Route[a]	Distances (tkm)			Trade flow (million tonnes)	Share of global gas trade	Energy intensity of transportat (MJ/kg)	References
	Onshore pipeline	Offshore pipeline	Sea freight				
UK – Belgium	0.55	0.235	0				Ecoinvent v3.2[59]
UK – Switzerland	0.7	0.235	0				Ecoinvent v3.2[59]
UK – Netherlands	0.65	0.235	0				Ecoinvent v3.2[59]
Netherlands-Austria	0.8	0	0				Ecoinvent v3.2[59]
Netherlands-France	0.2	0	0				Ecoinvent v3.2[59]
Netherlands – UK	0.25	0.235	0				Ecoinvent v3.2[59]
Norway – Belgium	0.1	0.65	0				Ecoinvent v3.2[59]
Norway – Switzerland	1.45	0.65	0				Ecoinvent v3.2[59]
Norway - Czechia	0.75	0.65	0				Ecoinvent v3.2[59]
Norway – Spain	1.55	0.65	0				Ecoinvent v3.2[59]
Germany – Austria	0.7	0	0				Ecoinvent v3.2[59]
Germany – Switzerland	0.85	0	0				Ecoinvent v3.2[59]
Germany – Poland	0.5	0	0				Ecoinvent v3.2[59]
**European domestic**	**0.7**	**0.235**	**0**		**5.28%**	**0.32**	Ecoinvent v3.2[59]
Algeria – Switzerland[b]	2.1	0.1	1.1				Ecoinvent v3.2[59]
Algeria - Spain[b]	1.2	0.1	0.6				Ecoinvent v3.2[59]
Algeria – France[b]	0.7	0.1	2.5				Ecoinvent v3.2[59]
Algeria – Italy[b]	1	0.1	1				Ecoinvent v3.2[59]
Algeria - UK[b]	0.7	0.1	2.1				Ecoinvent v3.2[59]
**North Africa-Europe**	**1**	**0.1**	**1.1**		**1.59%**	**0.79**	
Russia - Belgium	6.1	0	0				Ecoinvent v3.2[59]
Russia - Sweden	5.6	0	0				Ecoinvent v3.2[59]

Russia - Poland	3.7	0	0				Ecoinvent v3.2[59]
Russia - Italy	6.4	0	0				Ecoinvent v3.2[59]
**Russia-EU[c]**	**5.85**	**0**	**0**		**5.72%[c]**	**2.02**	Ecoinvent v3.2[59]
**USA (Ford Shale -Corpus Christi) – Europe (Rotterdam)[d]**	**0.1**	**0.02**	**9.6**		**0.48%**	**3.66**	Our Calculation
**Middle-East (Qatar)– Europe**	**0.6**	**0**	**10.0**		**1.04%**	**3.91**	Schori et al., 2012[79]
**Central and South America domestic[e]**	**0.6**	**0**	**0.4**		**4.2%**	**0.35**	Our Calculation
Alberta - Quebec (inside Canada)	**3800**	**9**	**0**				Ecoinvent v3.2[59]
USA (inside)	**1000**	**0**	**0**				Littlefield et al., 2019[80]
**USA and Canada domestic**	**1.3**	**0**	**0**		**24.6%**	**0.45**	Our Calculation
**Middle East domestic**	**0.2**	**0.02**	**0**		**14.2%**	**0.08**	Ecoinvent v3.2[59]
**Russia domestic[c]**	**2.5**	**0**	**0**		**14.6%**	**0.86**	Ecoinvent v3.2[59]
Russia (Siberia) – China (Shanghai)[f]	3	0	0		1.55%	2.42	Our Calculation
Qatar – China (Shanghai)	0.2	0	10.6[h]		0.44%	3.99	Our Calculation
Australia (North West Shelf) - China (Shanghai)[g]	0	0.12	5.7[h]		3.77%	2.15	Our Calculation
Malaysia (Bintulu) – China (Shanghai)[i]	0	0.15[i]	3,3[h]		2.04%	1.16	Our Calculation
**China (average)**	**1.4**	**0.1**	**4.1**		**7.8%**	**2.05**	Our Calculation
**Weighted average energy intensity of natural gas transport**					**100*%**	**0.76+/-0.18[j]**	

Notes: [a] Selected trade routes represent some of the most important natural gas pipelines and sea freight routes for liquified natural gas. We divide the trade routes into three transportation segments (onshore pipeline, offshore pipeline, freight by ships). To estimate the energy required in the transportation of natural gas, we first calculate median distances of natural gas across the transportation segments in each respective region. Estimates of median distances in the regions are indicated in bold letters in the Supplementary Table 6. To obtain the energy used in the transportation of natural gas in each respective region, we multiply median distances by the respective energy intensity coefficients, provided in Supplementary Table 3. We obtained the distances for most routes from the Ecoinvent v3.2 database, using the keywords: *natural gas, high pressure, import from "xxx"*.

[b] Natural gas from Algeria is transported to Europe in pipelines and in freight ships. The Ecoinvent database provides the joint average transportation distance from both transportation modes while considering the shares of natural gas that is transported in pipelines and in liquified natural gas (LNG) freight ships.

[c] In the calculations of energy requirements from trade routes that start in Russia, we also include the gas from other former countries of the Soviet Union (CIS countries).

[d] For the transportation of natural gas from the USA to Europe, we choose the Eagle Ford natural gas field, Texas and the main LNG export terminal in Corpus Christi[81], as Texas is the biggest producer of natural gas in the USA[82]. Only a small fraction of natural gas is produced offshore, in the Mexican Gulf, therefore the average distance of offshore pipelines in the respective transportation routes is estimated at 20 km, following the assumptions of the Ecoinvent database.

[e] For the transportation of natural gas in Latin America in onshore pipelines we (conservatively) estimate an average distance of 650 km. According to the BP table of major trade movements of natural gas, around 90% of natural gas in this region is moved by land. For the remaining 10% which is transported in LNG freight ships, we chose the representative trade route from Pampa Melchorita (Peru) to Manzanillo (Mexico) with a distance of 4700 km according to the "Sea Rates"[75] distance calculator.

[f] Estimated distance is taken from the documentation on the main line of the Power of Siberia 1 pipeline[83].

[g] For the transportation of natural gas from Australia, we chose the North West Shelf field, which is the main offshore source of natural gas in Australia. The local port Karratha is where natural gas is liquified and loaded onto ships[84].

[h] For the open sea trade routes, we used the application "Sea Rates" which calculates the distances between ports.

[i] Malaysia's LNG terminal that is based in Bintulu is the country's largest export hub for natural gas[85]. We used Google maps to estimate the distance of the offshore pipelines in Sarawak to be 150 km. The distance from Bintulu to Shanghai was estimated using "Sea Rates".

[j] We match the selected trade routes with the volume of transported natural gas in pipelines and ships in the "*Natural Gas Trade Movements 2019*" input and output tables from the BP's *"Statistical Review of World Energy 2020"*[77] According to BP's data tables on natural gas production and trade, the selected trade routes transport 80% of globally produced natural gas. We calculate relative shares of natural gas that is transported over a selected regional trade route and use them as relative weights to calculate the average global energy requirement for transporting natural gas, as shown in Equation 13 of the Methods. The standard error of our global intensity estimate is assumed to be the double of the standard deviation of the energy intensities in the selected trade routes.

Supplementary Table 14: Energy requirements of the global transportation of coal, estimated from the analysis of major global trade routes of coal.

Trade Route[a]	Distances (tkm)				Trade flow (EJ)[b]	Share of global coal production[c]	Energy intensity of transport (MJ/kg)	References
	Train	Barge	Lorry (16-32 tonnes)	Freight ship (bulk)				
**Domestic Transport**					**132.30**	**79.0%**	**0.15**	Ecoinvent v3.2[59]
Indonesia	0.15	0.15	0.05	0	5.87	3.50%	2.57	Ecoinvent v3.2[59]
Latin America	0.2	0	0.008	0	0.43	0.26%	1.03	Ecoinvent v3.2[59]
Australia	0.01	0.05	0	0	3.46	2.06%	0.59	Ecoinvent v3.2[59]
China	0.645	0.09	0.005	0	79.48	47.43%	1.03	Ecoinvent v3.2[59]
Europe	0.45	0.3	0	0	6.29	3.75%	1.36	Ecoinvent v3.2[59]
India	0.42	0	0.05	0	12.73	7.60%	1.41	Ecoinvent v3.2[59]
North America	0.38	0.03	0.005	0	12.26	7.31%	1.14	Ecoinvent v3.2[59]
Russia	0.8	0	0	0	5.02	3.0%	0.50	Ecoinvent v3.2[59]
South Africa	0.21	0	0.16	0	4.18	2.49%	2.18	Ecoinvent v3.2[59]
**International Transport**					**35.28**	**21.0%**	**0.89**	Ecoinvent v3.2[59]
Australia - Europe	0.25	0	0	23	6.3	0.39%	2.18	Ecoinvent v3.2[59]
Australia – East Asia (China)	0.25	0	0	9	1.71	3.76%	0.70	Ecoinvent v3.2[59]
Australia - Indonesia	0.25	0	0	5	0.15	0.01%	2.00	Ecoinvent v3.2[59]
Australia - Latin America	0.25	0	0	9	0	0.001%	1.12	Ecoinvent v3.2[59]
Australia - South Africa	0.25	0	0	12.5	0.83	0.03%	0.79	Ecoinvent v3.2[59]
Australia - India	0.25	0	0	10	6.47	0.50%	1.40	Ecoinvent v3.2[59]
Indonesia - East Asia China)	0.4	0	0	4	0.07	3.86%	0.85	Ecoinvent v3.2[59]
Indonesia - Europe	0.15	0.15	0.05	18	0	0.04%	1.40	Ecoinvent v3.2[59]
Indonesia - North America	0.15	0.15	0.05	18	2.61	0.01%	1.07	Ecoinvent v3.2[59]
Indonesia - India	0.15	0.15	0.05	4.6	0.25	1.56%	1.62	Ecoinvent v3.2[59]
Latin America – East Asia (China)	0.8	0	0.008	17	0.84	0.15%	0.50	Ecoinvent v3.2[59]
Latin America - Europe	0.8	0	0.008	9	0.03	0.50%	0.61	Ecoinvent v3.2[59]
Latin America - India	0.8	0	0.008	20	0.3	0.02%	1.27	Ecoinvent v3.2[59]

Latin America - North America	0.8	0	0.008	6	1.29	0.18%	2.11	Ecoinvent v3.2[59]
North America – East Asia (China)	0.38	0.03	0.005	12	0.96	0.77%	1.43	Ecoinvent v3.2[59]
North America - Europe	0.38	0.03	0.005	7	0	0.57%	2.57	Ecoinvent v3.2[59]
North America - India	0.38	0.03	0.005	16	6.47	0.24%	0.59	Ecoinvent v3.2[59]
North America - Latin America	0.38	0.03	0.005	9	0.23	3.86%	1.03	Ecoinvent v3.2[59]
North America - South Africa	0.38	0.03	0.005	14	2.73	0.14%	1.36	Ecoinvent v3.2[59]
Russia – East Asia (China)	2	0	0	2	2.54	1.63%	1.41	Ecoinvent v3.2[59]
Russia – Europe	2	0	0	3	0.22	1.52%	1.14	Ecoinvent v3.2[59]
Russia – India	2	0	0	9	0.16	0.13%	0.50	Ecoinvent v3.2[59]
South Africa – Europe	0.76	0	0.16	15.4	0.03	0.10%	2.18	Ecoinvent v3.2[59]
South Africa – Latin America	0.76	0	0.16	9.25	6.3	0.02%	1.63	Ecoinvent v3.2[59]
**Weighted average energy intensity of coal transport**					**167.58**	**100%**	**0.28 (0.18-0.48)[c]**	

Notes: [a] Selected trade routes represent the most relevant global trade routes of coal. We divided the trade routes into four transportation segments (train, lorry, freight by ship, and freight by barge), following the methodology from the Ecoinvent v3.2 database. Energy requirements for transportation using conveyor belt are neglected as they are one order of magnitude lower than the other means of transportation. We obtained the distances for domestic trade routes from the Ecoinvent v3.2 database, using the keywords: *market for hard coal*. For international trade routes, we used the keywords: *hard coal, import from "destination"*, also from the Ecoinvent v3.2 database.

[b] We match the selected international trade routes with the volume of transported coal obtained from "*Coal: Inter-area movements 2019*" input and output table from the BP's "*Statistical Review of World Energy 2020*"[77]. According to BP's data tables on global coal production and trade, selected trade routes represent 21% of globally produced coal. The remaining 79% of global coal production is transported and consumed domestically.

[c] We calculate relative shares of globally produced coal that is transported over a selected trade route and use them as relative weights to calculate the average energy use for the domestic and international transport of coal, as shown in Equation 13 of the Methods. For the lower-range estimate of energy intensity of global transportation of coal, we deduct from the average energy intensity the standard error of the energy intensity of domestic coal transport. For the upper range estimate we add to the median energy intensity of transporting coal, the standard error of the energy intensity of the international coal transport. The standard errors of the domestic and international energy intensity of coal transport is calculated as the standard deviation of the energy intensities of the selected trade routes.

Supplementary Table 15: Energy requirements of the global transportation of biomass, estimated from the data on global trade in biomass pellets.

Trade Route[a]	Distances (tkm)[b]			Trade flows (ktonne)	Share of global pellet production	Energy intensity of transport (MJ/kg)[c]
	Truck (<32 tonnes)	Train	Freight ship			
Canada – USA	0.1-0.4		0	**185**	**0.80%**	0.13-0.50
Canada – Europe	0.1-0.4		7	**1290**	**5.60%**	0.90-1.27
Canada – Japan	0.1-0.4		12	**80**	**0.35%**	1.45-1.82
Canada – South Korea	0.1-0.4		12	**50**	**0.21%**	1.45-1.82
USA – Europe	0.1-0.4		7	**4550**	**19.78%**	0.90-1.27
Europe domestic	0.1-0.4		0	**6680**	**29.04%**	0.13-0.50
Russia – Europe	0	2-4	3	**1115**	**4.85%**	2.85-5.34
Russia – South Korea	0	2-4	2	**70**	**0.30%**	2.74-5.26
Malaysia – South Korea	0.1-0.4		4	**115**	**0.50%**	0.57-0.94
Vietnam – South Korea	0.1-0.4		3	**600**	**2.60%**	0.46-0.83
Domestic	0.1-0.4		0	**8265**	**35.93%**	0.30-0.50
**Weighted average intensity of biomass transport**					**100%***	**0.36 - 0.74[c]**

Notes: [a] Trade routes as well as volumes of traded biomass are taken from the study by Junginger et al.[86] For transportation of biomass we could not apply the approach applied for other energy resources because biomass trade flows are not reported in the BP's "*Statistical Review of World Energy 2020*"report[77].

[b] For transportation across the open sea, we assume the same distances as in the corresponding trade routes from the case study of coal (see Supplementary Table 7). For distances of biomass transportation via a truck or train, we base the range of our assumptions on the estimates from life-cycle analysis literature*:* Fajardy and Mac Dowell, 2017[58], Fajardy and Mac Dowell, 2018[16], Pandur et al., 2015[36], Raugei and Lecissi, 2016[6].

[c] We calculate the relative shares of the global pellet production transported over a selected trade route and use them as relative weights to calculate the average energy intensity for biomass transportation, as shown in Equation 13 of the Methods. To obtain the energy used in the transportation of biomass in each respective trade route, we multiply the distances by the respective energy intensity coefficients, provided in Supplementary Table 3. Our estimates of average global energy intensity of biomass transport (0.36 – 0.74 MJ/kg) are comparable with the estimates from recent studies by Hanssen et al.,[87] who estimated 0.96 MJ/kg for the trade route from USA to Europe or from Raugei and Lecissi, 2016[6], who estimated 0.4 – 0.6 MJ/kg for the trade route from USA to UK.

Note on EROI_{FIN} dynamics in fossil fuels and biomass technologies

The EROI of energy technologies changes over time due to a range of factors. For depletable resources like oil and coal, energy requirements of extraction have increased over time due to declining resource abundance and are likely to continue to increase in the future[88,89]. This increase in energy requirements has led to a decline in the EROI of fossil fuels at the primary energy stage[65,90,91]. By contrast, research and innovation have improved the efficiency of energy conversion from primary to final energy, thereby increasing the amount of energy delivered per input of raw energy[92]. Previous studies find that values of the global EROI of fossil fuels at the final energy stage have not changed significantly over the 1990-2010 period, suggesting the two opposite tendencies have cancelled each other out[5,18].

Here, we model the EROI dynamics of fossil fuels and biomass technologies at the final energy stage by assuming the energy requirements associated with extraction will continue to increase over time, as shown in Equation 7 of the Methods. We model the changes to the energy conversion of fossil fuels by referring to the improvements in energy conversion efficiencies assumed in the REMIND model, summarised in Supplementary Table 9. As shown in Equation 10 of the Methods, an increase in the conversion efficiency (η_C) increases the amount of energy delivered per unit of energy invested, as it decreases the required input of the raw resource per unit of generated energy, thereby increasing the EROI of these technologies. For energy intensities of energy requirements along the energy supply chain (E_{TRA}, and E_{REF}), we do not explicitly model dynamic changes, as there are no assumptions about these processes in the IAMs. However, improvements in energy conversion efficiencies also decrease the energy requirements for maintaining the supply of raw fuels. Thus our EROI calculations broadly capture the underlying technological assumptions of the mitigation scenarios of low-carbon energy transition. Although we do not model the dynamics of all the processes, our range of energy intensities associated with the energy requirements can be read as best-case versus worst-case scenarios of energy requirements associated with the supply of raw energy fuels (see Supplementary Tables 3 and 4). The lower end of energy intensity parameters is based on the favourable assumptions that energy requirements in resource extraction, transportation, and processing or refining will decrease. Higher energy intensity parameters in turn assume a perspective of more costly operations of resource supply (e.g. longer trade routes, greater resource scarcity). The median energy intensity parameters describe a balanced, middle-of-the road scenario.

Note on EROI dynamics of wind and solar power

Historically, the EROIs of renewables have increased due to improvements in energy efficiency and declining energy requirements, achieved through technological innovation and the upscaling of power plant production[20,93].

To model the dynamic evolution of the EROI of PV and wind power, we estimate the effects of technological innovation on the energy requirements of the construction, and operation and maintenance of these technologies, using the "energetic experience curves" approach by Steffen et al.[18]. The experience curves approach is a well-established methodology for quantifying the improvement in technologies with their increasing deployment[20,94,95]. The underlying assumption of this approach is that the performance of a given technology increases proportionally to its uptake by society.

In our case study, the energy requirements of the technology decrease proportionally to the cumulative installed capacity of the technology in relation to the present-day energy intensities of construction, and operation and maintenance $\theta_{CON,0}$ and $\theta_{O\&M,0}$, as shown in Supplementary Equations 1 and 2. The rate of improvement is determined by the experience rate (b), which we calculate from the "invested energy data" provided by Steffen et al., in Fig. 1 of their article. We obtain the present-day energy intensities of construction and operation per installed unit of power, by dividing the energy requirements for operation and the requirements of construction by the power capacity, as described in Supplementary Equations 3 and 4.

$$\theta_{CON}(t) = \theta_{CON,0} \cdot \left(\frac{P_{CUM}(t)}{P_{CUM,0}}\right)^{-b} \quad (1)$$

$$\theta_{O\&M}(t) = \theta_{O\&M,0} \cdot \left(\frac{P_{CUM}(t)}{P_{CUM,0}}\right)^{-b} \quad (2)$$

$$\theta_{CON,0} = \frac{0.9 \cdot (1 - \alpha_{tech}) \cdot \mathrm{CF}_0 \cdot \tau}{EROI_o} \quad (3)$$

$$\theta_{O\&M,0} = \frac{\alpha_{tech} \cdot \mathrm{CF}_0}{EROI_0} \quad (4)$$

We do not apply the experience curve approach to estimate improvements in energy conversion efficiencies, as these improvements and the effects on the EROI of PV and wind power are already endogenously accounted for in the scenario data of generated energy.

By calculating the energy intensity of construction and operation of renewables, over time, we can estimate the future energy requirements of PV and wind power, as well as the EROIs of these technologies as shown in Supplementary Equations 5 and 6.

$$E_{CON}(t) = \theta_{CON}(t) \cdot P_{NEW}(t) \quad (5)$$

$$E_{OPR} = \theta_{OPR}(t) \cdot P(t) \quad (6)$$

Finally, after including the energy requirements associated with decommissioning (see Equation 18 of the Methods), we obtain the equation for calculating the EROIs of PV and wind, as shown below:

$$\text{EROI(t)} = \frac{E_{GEN}}{0.9 \cdot \theta_{CAP}(t) \cdot P_{NEW}(t) + \theta_{O\&M}(t) \cdot P(t) + 0.1 \cdot \theta_{CAP}(t-\tau) \cdot P_{NEW}(t-\tau))} \quad (7)$$

However, the historical trend of improving EROIs of renewables may slow down or even reverse in the future due to several factors. EROI improvements may slow down with decreasing resource density (e.g. average wind power density), as the best sites are developed first and there are fewer sites with abundant resources[96]. Moreover, the EROI of renewables may decline as their share in the energy mix increases due to intermittent variability in the energy supply from renewables, depending on the environmental conditions[97]. During favourable conditions, the electricity generated from renewables may exceed the electricity demand, causing the overcapacity of renewable energy infrastructure as well as of the "conventional power plants" to be temporarily switched-off the energy grid. By contrast, to ensure reliable energy supply during unfavourable conditions, when renewables do not meet the electricity demand, the electric grid will have to be reinforced with additional power plants (overcapacity) and energy storage solutions[98,99], both of which are energetically costly and therefore decrease the EROI of the overall energy system[2].

Our calculations only allow for a rough estimation of energy investment in the construction, and operation and maintenance of intermittent energy delivery. As wind and solar power become more important sources in the energy mix, such intermittent energy sources will require additional energy investments due to energy losses from excess power supply (curtailment) and from the energy requirements of storage[,97].

We account for factors that may lead to a decline in the EROI of renewables (e.g. overcapacity and energy storage) to the extent that these effects are represented in the data of mitigation scenarios for energy generation. Some scenarios take into consideration the need for additional installed power capacity. The storage solutions in the mitigation scenarios are explicitly only represented by hydrogen from electrolysis[98]. For a description of our method of calculating the EROI of hydrogen from electrolysis, see the "Note on energy requirements of hydrogen from hydrolysis" (in Supplementary Information).

We only account for invested energy for the production side of the energy system, but not the energy required for the supporting energy infrastructure, like smart power-grids.

Note on energy requirements of hydrogen from electrolysis

Most of the mitigation scenarios analysed in this study include hydrogen in their basket of low-carbon energy carriers. These scenarios are LED, S1-I, S1-G, S1-M, S1-R, S2-G, S2-M, S2-R, S5-G, and S5-R. The hydrogen energy conversion technologies include: hydrogen from biomass, hydrogen from biomass with CCS, and hydrogen from electrolysis. Our calculation of energy requirements of hydrogen from biomass follows the general approach for biomass technologies, described in the Methods section: "Energy requirements of fossil fuels and biomass technologies".

To estimate energy requirements of hydrogen generated from electrolysis, we divide the amount of generated hydrogen by the EROI of hydrogen from electrolysis, as shown below:

$$E_{REQ} = \frac{E_{GEN}}{EROI_{H2}} \quad (8)$$

In estimating the EROI of hydrogen from electrolysis, we follow the approach of Pellow et al.[62]. Here, EROI of hydrogen from electrolysis depends on the efficiency of energy conversion from electricity to hydrogen (η_{sys}), and the energy embodied in the production of the hydrogen fuel cell, reflected in the ESOI, alongside the energy requirements associated with the generation of electricity in the grid (EROI_e), as shown in Supplementary Equation 9.

$$\mathrm{EROI}_{H2} = \frac{\eta_{sys}}{\frac{1}{\mathrm{EROI}_e} + \frac{1}{\mathrm{ESOI}}} \quad (9)$$

Here, ESOI stands for "Energy stored on energy invested", defined as a ratio between the energy stored in a fuel cell over its lifetime and the energy that is required to manufacture and maintain the fuel cell. We calculate the EROI of electricity by summing the total amount of generated electricity, across all of the electricity generation technologies, and dividing it by the sum of the total energy requirements of electricity generation.

$$\mathrm{EROI}_e = \frac{\sum_e E_{GEN,e}}{\sum_e E_{REQ,e}} \quad (10)$$

The system efficiency parameter depends on the efficiencies of the electrolyser (η_{lyz}), fuel cell efficiency (η_{FC}), and hydrogen compression (η_{comp}) as in Supplementary Equation 11[62]:

$$\eta_{sys} = \frac{\eta_{lyz}\eta_{FC}}{1 + \eta_{lyz}(\frac{1}{\eta_{comp}} - 1)} \quad (11)$$

In our estimates of the EROI of hydrogen from electrolysis, we conducted a sensitivity analysis on the efficiency parameters, using three assumptions: median, optimistic, and very optimistic. Parameter values were taken from Pellow et al.[62]. The median assumptions resemble the efficiency parameters of best available technologies, whereas optimistic and very optimistic parameters represent different assumptions on the future technological innovation of hydrogen. Very optimistic parameters represent efficiencies that are close to their thermodynamic limit. The values of the efficiency parameters are provided together with the ESOI calculations in Supplementary Table 9.

Carbon intensities of the four energy carriers in the mitigation pathways

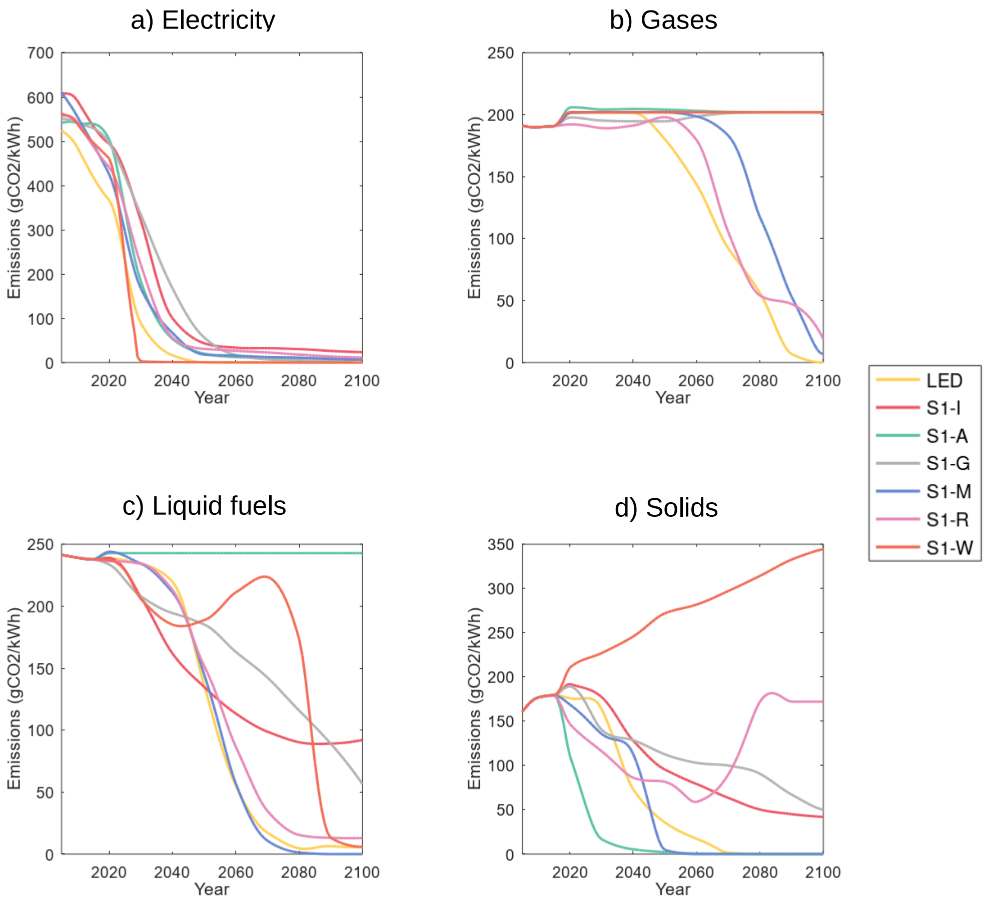

Supplementary Figure 8: Carbon intensities of energy carriers in the first group of pathways used in our study. Electricity undergoes the fastest decarbonisation amongst the four energy carriers across the scenarios. Gases, liquids, and solids are fully decarbonised only in some pathways (LED, S1-M, S2-M) whereas in others these carriers are either partially decarbonised or their carbon intensities even remain constant over time. These carriers are decarbonised in pathways that switch from fossil fuels to biomass, as their primary raw energy source, changing the traditional energy carriers of oil (petroleum), natural gas, and coal, for the alternatives such as biofuels, synthetic “biogases”, and biomass pellets. Moreover, some scenarios (S1-M, S2-M, S1-R, S2-R, S5-R) decarbonise the energy services provided by liquid fuels by transitioning from oil to hydrogen-powered engines. The pathways that do not fully decarbonise all four energy carriers accomplish the emissions reductions by downsizing the use of the carriers of high carbon intensity, substituting them with decarbonised energy carriers, or by offsetting emissions using negative emissions from BECCS. The example of substation between carriers would be a transition from internal combustion engine vehicles to electric vehicles. For example, the S1-A and S2-A scenarios accomplish a rapid decarbonisation by assuming energy transition towards an energy system based on low-carbon electricity, thereby downscaling the importance of other carriers in the energy system. Carbon intensities of the second group of pathways are shown in Supplementary Figure 7.

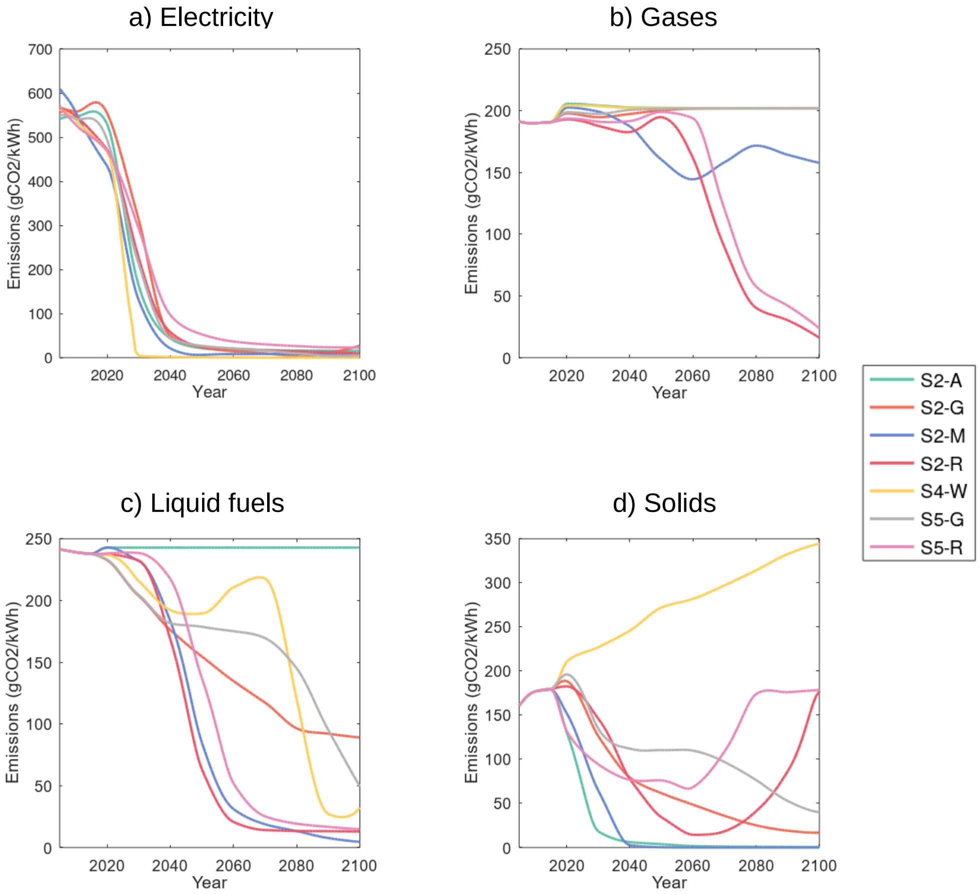

Supplementary Figure 9: Carbon intensities of energy carriers in the second group of pathways used in our study.

Supplementary References

1. Raugei, M. Net energy analysis must not compare apples and oranges. *Nat. Energy* **4**, 86–88 (2019).
2. Castro, C. de & Capellán-Pérez, I. Standard , Point of Use , and Extended Energy Return. *Energies* **13**, (2020).
3. Song, Y. S., Youn, J. R. & Gutowski, T. G. Life cycle energy analysis of fiber-reinforced composites. *Compos. Part A Appl. Sci. Manuf.* **40**, 1257–1265 (2009).
4. Guan, J., Zhang, Z. & Chu, C. Quantification of building embodied energy in China using an input-output-based hybrid LCA model. *Energy Build.* **110**, 443–452 (2016).
5. Brockway, P. E., Owen, A., Brand-Correa, L. I. & Hardt, L. Estimation of global final stage energy-return-on-investment for fossil fuels with comparison to renewable energy sources. *Nat. Energy* **4**, (2019).
6. Raugei, M. & Leccisi, E. A comprehensive assessment of the energy performance of the full range of electricity generation technologies deployed in the United Kingdom. *Energy Policy* **90**, 46–59 (2016).
7. Leccisi, E., Raugei, M. & Fthenakis, V. The energy and environmental performance of ground-mounted photovoltaic systems - A timely update. *Energies* **9**, (2016).
8. Ferroni, F., Guekos, A. & Hopkirk, R. J. Further considerations to: Energy Return on Energy Invested (ERoEI) for photovoltaic solar systems in regions of moderate insolation. *Energy Policy* **107**, 498–505 (2017).
9. Raugei, M. *et al.* Energy Return on Energy Invested (ERoEI) for photovoltaic solar systems in regions of moderate insolation: A comprehensive response. *Energy Policy* **102**, 377–384 (2017).
10. Kubiszewski, I., Cleveland, C. J. & Endres, P. K. Meta-analysis of net energy return for wind power systems. *Renew. Energy* **35**, 218–225 (2010).
11. Hall, C. A. S., Lambert, J. G. & Balogh, S. B. EROI of different fuels and the implications for society. *Energy Policy* **64**, 141–152 (2014).
12. Sgouridis, S., Carbajales-Dale, M., Csala, D., Chiesa, M. & Bardi, U. Comparative net energy analysis of renewable electricity and carbon capture and storage. *Nat. Energy* **4**, 456–465 (2019).
13. King, L. C. & Van Den Bergh, J. C. J. M. Implications of net energy-return-on-investment for a low-carbon energy transition. *Nat. Energy* **3**, 334–340 (2018).
14. Raugei, M., Fullana-i-Palmer, P. & Fthenakis, V. The energy return on energy investment (EROI) of photovoltaics: Methodology and comparisons with fossil fuel life cycles. *Energy Policy* **45**, 576–582 (2012).
15. Murphy, D. J. & Hall, C. A. S. Year in review-EROI or energy return on (energy) invested. *Ann. N. Y. Acad. Sci.* **1185**, 102–118 (2010).
16. Fajardy, M. & Mac Dowell, N. The energy return on investment of BECCS: Is BECCS a threat to energy security? *Energy Environ. Sci.* **11**, 1581–1594 (2018).
17. Atlason, R. S. & Unnthorsson, R. Hot water production improves the energy return on

investment of geothermal power plants. *Energy* **51**, 273–280 (2013).

18. Steffen, B., Hischier, D. & Schmidt, T. S. Historical and projected improvements in net energy performance of power generation technologies. *Energy Environ. Sci.* **11**, 3524–3530 (2018).

19. Limpens, G. & Jeanmart, H. Electricity storage needs for the energy transition: An EROI based analysis illustrated by the case of Belgium. *Energy* **152**, 960–973 (2018).

20. Louwen, A., Van Sark, W. G. J. H. M., Faaij, A. P. C. & Schropp, R. E. I. Re-assessment of net energy production and greenhouse gas emissions avoidance after 40 years of photovoltaics development. *Nat. Commun.* **7**, 1–9 (2016).

21. Dupont, E., Koppelaar, R. & Jeanmart, H. Global available wind energy with physical and energy return on investment constraints. *Appl. Energy* **209**, 322–338 (2018).

22. Sathre, R. *et al.* Life-cycle net energy assessment of large-scale hydrogen production via photoelectrochemical water splitting. *Energy Environ. Sci.* **7**, 3264–3278 (2014).

23. Sathre, R. *et al.* Opportunities to improve the net energy performance of photoelectrochemical water-splitting technology. *Energy Environ. Sci.* **9**, 803–819 (2016).

24. Hacatoglu, K., Rosen, M. A. & Dincer, I. Comparative life cycle assessment of hydrogen and other selected fuels. *Int. J. Hydrogen Energy* **37**, 9933–9940 (2012).

25. Yaritani, H. & Matsushima, J. Analysis of the Energy Balance of Shale Gas Development. *Energies* **7**, 2207–2227 (2014).

26. Gagnon, N., Hall, C. A. S. & Brinker, L. A preliminary investigation of energy return on energy investment for global oil and gas production. *Energies* **2**, 490–503 (2009).

27. Feng, J., Feng, L. & Wang, J. Analysis of point-of-use energy return on investment and net energy yields from China's conventional fossil fuels. *Energies* **11**, 1–21 (2018).

28. Uusitalo, V., Leino, M., Kasurinen, H. & Linnanen, L. Transportation biofuel efficiencies from cultivated feedstock in the boreal climate zone: Case Finland. *Biomass and Bioenergy* **99**, 79–89 (2017).

29. Felder, R. & Dones, R. Evaluation of ecological impacts of synthetic natural gas from wood used in current heating and car systems. *Biomass and Bioenergy* **31**, 403–415 (2007).

30. Brandt, A. R. Oil depletion and the energy efficiency of oil production: The case of California. *Sustainability* **3**, 1833–1854 (2011).

31. Hall, C. A. S., Balogh, S. & Murphy, D. J. R. What is the minimum EROI that a sustainable society must have? *Energies* **2**, 25–47 (2009).

32. Kong, Z. *et al.* EROI analysis for direct coal liquefaction without and with CCS: The case of the Shenhua DCL Project in China. *Energies* **8**, 786–807 (2015).

33. Murphy, D. J., Hall, C. A. S., Dale, M. & Cleveland, C. Order from chaos: A preliminary protocol for determining the EROI of fuels. *Sustainability* **3**, 1888–1907 (2011).

34. De Castro, C., Carpintero, Ó., Frechoso, F., Mediavilla, M. & de Miguel, L. J. A top-down approach to assess physical and ecological limits of biofuels. *Energy* **64**, 506–512 (2014).

35. Mandade, P. & Shastri, Y. Multi-objective optimization of lignocellulosic feedstock selection for ethanol production in India. *J. Clean. Prod.* **231**, 1226–1234 (2019).

36. Pandur, Z., Šušnjar, M., Zorić, M., Nevečerel, H. & Horvat, D. Energy Return on Investment (EROI) of Different Wood Products. *Precious For. - Precious Earth* (2015). doi:10.5772/61144

37. Dale, M., Krumdieck, S. & Bodger, P. Global energy modelling - A biophysical approach (GEMBA) Part 1: An overview of: Biophysical economics. *Ecol. Econ.* **73**, 152–157 (2012).

38. Dale, M., Krumdieck, S. & Bodger, P. Global energy modelling - A biophysical approach (GEMBA) Part 2: Methodology. *Ecol. Econ.* **73**, 158–167 (2012).

39. Raugei, M., Leccisi, E., Fthenakis, V., Escobar Moragas, R. & Simsek, Y. Net energy analysis and life cycle energy assessment of electricity supply in Chile: Present status and future scenarios. *Energy* **162**, 659–668 (2018).

40. Aguirre-Villegas, H. A. & Benson, C. H. Case history of environmental impacts of an Indonesian coal supply chain. *J. Clean. Prod.* **157**, 47–56 (2017).

41. Trainer, T. Estimating the EROI of whole systems for 100% renewable electricity supply capable of dealing with intermittency. *Energy Policy* **119**, 648–653 (2018).

42. Capellán-Pérez, I., de Castro, C. & Miguel González, L. J. Dynamic Energy Return on Energy Investment (EROI) and material requirements in scenarios of global transition to renewable energies. *Energy Strateg. Rev.* **26**, 100399 (2019).

43. Sgouridis, S., Csala, D. & Bardi, U. The sower's way: quantifying the narrowing net-energy pathways to a global energy transition. *Environ. Res. Lett.* **11**, 094009 (2016).

44. IPCC, 2018: Global warming of 1.5°C. An IPCC Special Report on the impacts of global warming of 1.5°C above pre-industrial levels and related global greenhouse gas emission pathways, in the context of strengthening the global response to the threat of climate change, sustainable development, and efforts to erradicate poverty. [Masson-Delmotte, V., P. Zhai, H.-O. Pörtner, D. Roberts, J. Skea, P.R. Shukla, A. Pirani, W. Moufouma-Okia, C. Péan, R. Pidcock, S. Connors, J.B.R. Matthews, Y. Chen, X. Zhou, M.I. Gomis, E. Lonnoy, T. Maycock, M. Tignor, and T. Waterfield (eds.)]. In Press.

45. Grubler, A. *et al.* A low energy demand scenario for meeting the 1.5 °c target and sustainable development goals without negative emission technologies. *Nat. Energy* **3**, 515–527 (2018).

46. Rogelj, J. *et al.* Scenarios towards limiting global mean temperature increase below 1.5°C. *Nat. Clim. Chang.* **8**, 325–332 (2018).

47. Riahi, K. *et al.* The Shared Socioeconomic Pathways and their energy, land use, and greenhouse gas emissions implications: An overview. *Glob. Environ. Chang.* **42**, 153–168 (2017).

48. van Vuuren, D. P. *et al.* Energy, land-use and greenhouse gas emissions trajectories under a green growth paradigm. *Glob. Environ. Chang.* **42**, 237–250 (2017).

49. Fricko, O. *et al.* The marker quantification of the Shared Socioeconomic Pathway 2: A middle-of-the-road scenario for the 21st century. *Glob. Environ. Chang.* **42**, 251–267 (2017).

50. Calvin, K. *et al.* The SSP4: A world of deepening inequality. *Glob. Environ. Chang.* **42**, 284–296 (2017).

51. Kriegler, E. *et al.* Fossil-fueled development (SSP5): An energy and resource intensive scenario for the 21st century. *Glob. Environ. Chang.* **42**, 297–315 (2017).

52. Luderer, G. *et al. Description of the REMIND model (Version 1.5)*. (2013).

53. IAMC. Documentation, Energy conversion - REMIND (Electricity). (2019).

54. Larson, E. *et al.* Chapter 12 - Fossil Energy. in *Global Energy Assessment: Toward a Sustainable Future* 901–992 (2012). doi:https://doi.org/10.1017/CBO9780511793677.018

55. IPCC. Plant efficiency and fuel switching. *IPCC Fourth Assessment Report: Climate Change 2007.* (2007). Available at: https://archive.ipcc.ch/publications_and_data/ar4/wg3/en/ch4s4-4-3-1.html.

56. Meili, C., Niels, J. & Wenzel, P. *Life cycle inventories of long-distance transport of crude oil*. (2018).

57. Jing, L. *et al.* Carbon intensity of global crude oil refining and mitigation potential. *Nat. Clim. Chang.* **10**, 526–532 (2020).

58. Fajardy, M. & Mac Dowell, N. Can BECCS deliver sustainable and resource efficient negative emissions? *Energy Environ. Sci.* **10**, 1389–1426 (2017).

59. Ecoinvent. Ecoinvent Centre for Life Cycle inventories. *Ecoinvent LCI database version 3.2* (2020). Available at: https://www.ecoinvent.org/. (Accessed: 5th June 2021)

60. CAPP. What is the Average Life Span of a Well? *What is the Average Life Span of a Well* (2021). Available at: https://www.capp.ca/explore/life-cycle-of-a-well/.

61. Arvesen, A., Luderer, G., Pehl, M., Bodirsky, B. L. & Hertwich, E. G. Deriving life cycle assessment coefficients for application in integrated assessment modelling. *Environ. Model. Softw.* **99**, 111–125 (2018).

62. Pellow, M. A., Emmott, C. J. M., Barnhart, C. J. & Benson, S. M. Hydrogen or batteries for grid storage? A net energy analysis. *Energy Environ. Sci.* **8**, 1938–1952 (2015).

63. Lambert, J., Hall, C., Balogh, S., Poisson, A. & Gupta, A. *EROI of Global Energy Resources. Preliminary Status and Trends*. (2012).

64. Weißbach, D. *et al.* Energy intensities, EROIs (energy returned on invested), and energy payback times of electricity generating power plants. *Energy* **52**, 210–221 (2013).

65. Hu, Y., Hall, C. A. S., Wang, J., Feng, L. & Poisson, A. Energy Return on Investment (EROI) of China's conventional fossil fuels: Historical and future trends. *Energy* **54**, 352–364 (2013).

66. Yáñez, E., Ramírez, A., Uribe, A., Castillo, E. & Faaij, A. Unravelling the potential of energy efficiency in the Colombian oil industry. *J. Clean. Prod.* **176**, 604–628 (2018).

67. Cleveland, C. J. & O'Connor, P. A. Energy return on investment (EROI) of oil shale. *Sustainability* **3**, 2307–2322 (2011).

68. Poisson, A. & Hall, C. A. S. Time series EROI for Canadian oil and gas. *Energies* **6**, 5940–5959 (2013).

69. Guilford, M. C., Hall, C. A. S., O'Connor, P. & Cleveland, C. J. A new long term assessment of energy return on investment (EROI) for U.S. oil and gas discovery and production. *Sustainability* **3**, 1866–1887 (2011).

70. Sell, B., Murphy, D. & Hall, C. A. S. Energy return on energy invested for tight gas wells in the appalachian basin, United States of America. *Sustainability* **3**, 1986–2008 (2011).

71. Moriarty, P. & Honnery, D. Review: Assessing the climate mitigation potential of biomass. *AIMS Energy* **5**, 20–38 (2016).

72. Hall, C. A. S., Dale, B. E. & Pimentel, D. Seeking to understand the reasons for different energy return on investment (EROI) estimates for biofuels. *Sustainability* **3**, 2413–2432 (2011).

73. Voinov, A., Arodudu, O., Van Duren, I., Morales, J. & Qin, L. Estimating the potential of roadside vegetation for bioenergy production. *J. Clean. Prod.* **102**, 213–225 (2015).

74. NRCAN. Moving crude oil in Canada - Key existing pipelines in Canada. *Crude oil facts* (2020). Available at: https://www.nrcan.gc.ca/science-data/data-analysis/energy-data-analysis/energy-facts/crude-oil-facts/20064#L9.

75. Sea Rates. Shipping Distances & Time Calculator. (2021). Available at: https://www.searates.com/services/distances-time/.

76. Global Energy Monitor. Eastern Siberia-Pacific Ocean Oil Pipeline. *Global Fossil Infrastructure Tracker* (2021). Available at: https://www.gem.wiki/Eastern_Siberia–Pacific_Ocean_Oil_Pipeline.

77. BP. *Statistical Review of World Energy 2020*. (2020).

78. Strogen, B. & Horvath, A. Greenhouse Gas Emissions from the Construction, Manufacturing, Operation, and Maintenance of U.S. Distribution Infrastructure for Petroleum and Biofuels. *J. Infrastruct. Syst.* **19**, 371–383 (2013).

79. Schori, S. *et al. Life Cycle Inventory of Natural Gas Supply*. (2012).

80. Littlefield, J. *et al. Life Cycle Analysis of Natural Gas Extraction and Power Generation*. (2019).

81. Global Energy Monitor. Texas and Fracking. *FrackSwarm* (2012). Available at: https://www.gem.wiki/Texas_and_fracking#Corpus_Christi_LNG. (Accessed: 22nd May 2021)

82. EIA. U.S. dry natural gas production by state in 2019. *Natural gas explained* (2021). Available at: https://www.eia.gov/energyexplained/natural-gas/where-our-natural-gas-comes-from.php.

83. Gazprom. Power of Siberia: The largest gas transmission system in Russia's East. *Projects* (2021). Available at: https://www.gazprom.com/projects/power-of-siberia/. (Accessed: 22nd May 2021)

84. Woodside. North West Shelf. *Australian Operations* (2021). Available at: https://www.woodside.com.au/what-we-do/australian-operations/north-west-shelf. (Accessed: 22nd May 2021)

85. Monitor, G. E. Tiga Malaysia LNG Terminal. *Fossil Infrastructure Tracker* (2021). Available at: https://www.gem.wiki/Tiga_Malaysia_LNG_Terminal. (Accessed: 22nd May 2021)

86. Junginger, H. M. *et al.* The future of biomass and bioenergy deployment and trade: a synthesis of 15 years IEA Bioenergy Task 40 on sustainable bioenergy trade. *Biofuels, Bioprod. Biorefining* **13**, 247–266 (2019).

87. Hanssen, S. V., Duden, A. S., Junginger, M., Dale, V. H. & van der Hilst, F. Wood pellets, what else? Greenhouse gas parity times of European electricity from wood pellets produced in the south-eastern United States using different softwood feedstocks. *GCB Bioenergy* **9**, 1406–1422 (2017).

88. Dale, M., Krumdieck, S. & Bodger, P. A dynamic function for energy return on investment. *Sustainability* **3**, 1972–1985 (2011).

89. Sgouridis, S., Carbajales-Dale, M., Csala, D., Chiesa, M. & Bardi, U. Comparative net energy analysis of renewable electricity and carbon capture and storage. *Nat. Energy* **4**, 456–465 (2019).

90. Heun, M. K. & de Wit, M. Energy return on (energy) invested (EROI), oil prices, and energy transitions. *Energy Policy* **40**, 147–158 (2012).

91. Cleveland, C. J. Net energy from the extraction of oil and gas in the United States. *Energy* **30**, 769–782 (2005).

92. EEA. Efficiency of public conventional thermal power production in Europe. *Indicator Assesment* (2018). Available at: https://www.eea.europa.eu/data-and-maps/indicators/efficiency-of-conventional-thermal-electricity-generation-4/assessment-2. (Accessed: 5th June 2021)

93. Diesendorf, M. & Wiedmann, T. Implications of Trends in Energy Return on Energy Invested (EROI) for Transitioning to Renewable Electricity. *Ecol. Econ.* **176**, 106726 (2020).

94. Creutzig, F. *et al.* The underestimated potential of solar energy to mitigate climate change. *Nat. Energy* **2**, (2017).

95. Rubin, E. S., Azevedo, I. M. L., Jaramillo, P. & Yeh, S. A review of learning rates for electricity supply technologies. *Energy Policy* **86**, 198–218 (2015).

96. Miller, L. M. & Keith, D. W. Observation-based solar and wind power capacity factors and power densities. *Environ. Res. Lett.* **13**, (2018).

97. Barnhart, C. J., Dale, M., Brandt, A. R. & Benson, S. M. The energetic implications of curtailing versus storing solar- and wind-generated electricity. *Energy Environ. Sci.* **6**, 2804–2810 (2013).

98. Sullivan, P., Krey, V. & Riahi, K. Impacts of considering electric sector variability and reliability in the MESSAGE model. *Energy Strateg. Rev.* **1**, 157–163 (2013).

99. Delucchi, M. A. & Jacobson, M. Z. Providing all global energy with wind, water, and solar power, Part II: Reliability, system and transmission costs, and policies. *Energy Policy* **39**, 1170–1190 (2011).
